# Completeness of Reporting in Diet- and Nutrition-Related Randomized Controlled Trials and Systematic Reviews With Meta-Analysis: Protocol for 2 Independent Meta-Research Studies

**DOI:** 10.2196/43537

**Published:** 2023-03-23

**Authors:** Flávia Silva, Amanda Rodrigues Amorim Adegboye, Carl Lachat, Cintia Curioni, Fabio Gomes, Gary S Collins, Gilberto Kac, Jennifer Anne de Beyer, Jonathan Cook, Leila Cheikh Ismail, Matthew Page, Neha Khandpur, Sarah Lamb, Sally Hopewell, Shona Kirtley, Solange Durão, Colby J Vorland, Michael M Schlussel

**Affiliations:** 1 Nutrition Department Universidade Federal de Ciências da Saúde de Porto Alegre - UFCSPA Porto Alegre Brazil; 2 Centre for Healthcare Research Coventry University Coventry United Kingdom; 3 Department of Food Technology, Safety and Health Ghent University Ghent Belgium; 4 Department of Nutrition in Public Health State University of Rio de Janeiro Rio de Janeiro Brazil; 5 Pan-American Health Organization World Health Organization Washington, WA United States; 6 UK EQUATOR Centre Centre for Statistics in Medicine, Nuffield Department of Orthopaedics, Rheumatology and Musculoskeletal Sciences University of Oxford Oxford United Kingdom; 7 Nutritional Epidemiology Observatory, Department of Social and Applied Nutrition Institute of Nutrition Josué de Castro Federal University of Rio de Janeiro Rio de Janeiro Brazil; 8 Oxford Clinical Trials Research Unit Centre for Statistics in Medicine, Nuffield Department of Orthopaedics, Rheumatology and Musculoskeletal Sciences University of Oxford Oxford United Kingdom; 9 Department of Clinical Nutrition and Dietetics College of Health Sciences Sharjah United Arab Emirates; 10 Methods in Evidence Synthesis Unit School of Public Health and Preventive Medicine Monash University Melbourne Australia; 11 Division of Human Nutrition and Health Wageningen University and Research Wageningen Netherlands; 12 College of Medicine and Health University of Exeter Exeter United Kingdom; 13 Cochrane South Africa South African Medical Research Council South Africa Cape Town South Africa; 14 Department of Epidemiology and Biostatistics Indiana University School of Public Health-Bloomington Bloomington, IN United States

**Keywords:** nutrition, diet, randomized controlled trials, CONSORT, TIDieR, PRISMA, spin, risk, bias, research, intervention, literature, limitations, PubMed

## Abstract

**Background:**

Journal articles describing randomized controlled trials (RCTs) and systematic reviews with meta-analysis of RCTs are not optimally reported and often miss crucial details. This poor reporting makes assessing these studies’ risk of bias or reproducing their results difficult. However, the reporting quality of diet- and nutrition-related RCTs and meta-analyses has not been explored.

**Objective:**

We aimed to assess the reporting completeness and identify the main reporting limitations of diet- and nutrition-related RCTs and meta-analyses of RCTs, estimate the frequency of reproducible research practices among these RCTs, and estimate the frequency of distorted presentation or spin among these meta-analyses.

**Methods:**

Two independent meta-research studies will be conducted using articles published in PubMed-indexed journals. The first will include a sample of diet- and nutrition-related RCTs; the second will include a sample of systematic reviews with meta-analysis of diet- and nutrition-related RCTs. A validated search strategy will be used to identify RCTs of nutritional interventions and an adapted strategy to identify meta-analyses in PubMed. We will search for RCTs and meta-analyses indexed in 1 calendar year and randomly select 100 RCTs (June 2021 to June 2022) and 100 meta-analyses (July 2021 to July 2022). Two reviewers will independently screen the titles and abstracts of records yielded by the searches, then read the full texts to confirm their eligibility. The general features of these published RCTs and meta-analyses will be extracted into a research electronic data capture database (REDCap; Vanderbilt University). The completeness of reporting of each RCT will be assessed using the items in the CONSORT (Consolidated Standards of Reporting Trials), its extensions, and the TIDieR (Template for Intervention Description and Replication) statements. Information about practices that promote research transparency and reproducibility, such as the publication of protocols and statistical analysis plans will be collected. There will be an assessment of the completeness of reporting of each meta-analysis using the items in the Preferred Reporting Items for Systematic reviews and Meta-Analysis (PRISMA) statement and collection of information about spin in the abstracts and full-texts. The results will be presented as descriptive statistics in diagrams or tables. These 2 meta-research studies are registered in the Open Science Framework.

**Results:**

The literature search for the first meta-research retrieved 20,030 records and 2182 were potentially eligible. The literature search for the second meta-research retrieved 10,918 records and 850 were potentially eligible. Among them, random samples of 100 RCTs and 100 meta-analyses were selected for data extraction. Data extraction is currently in progress, and completion is expected by the beginning of 2023.

**Conclusions:**

Our meta-research studies will summarize the main limitation on reporting completeness of nutrition- or diet-related RCTs and meta-analyses and provide comprehensive information regarding the particularities in the reporting of intervention studies in the nutrition field.

**International Registered Report Identifier (IRRID):**

DERR1-10.2196/43537

## Introduction

### Background

Nutrition research provides evidence to support public health policies, advances in clinical practice, and improved quality of life for patients. Reporting diet- and nutrition-related randomized controlled trials (RCTs) requires particular attention to field-specific methodological aspects, such as baseline dietary patterns, food intake assessment instruments, and statistical analysis methods [1-3]. Strategies to deal with methodological limitations and increase transparency in nutrition research include prioritizing large and long-term RCTs, pre-registering protocols and statistical analyses, clearly defining and standardizing methods for data collection and outcomes assessment, and promoting data sharing [4-7]. Guidelines on how to handle sensitive issues, such as public-private partnerships and working with industry, are available [8,9]. All of these recommendations, however, require complete and transparent reporting, which is also crucial when these nutrition- or diet-related RCTs are pooled in a systematic review or meta-analysis. Clearly specified methods for identifying, selecting, and appraising primary studies included in systematic reviews are essential to a systematic review being of high enough quality to inform clinical practice [10,11]. Systematic reviews are the cornerstones of therapeutic evaluation as they assess and grade the available body of evidence on interventions.

Inappropriate reporting of research often means an important and avoidable source of that research wasted [12], with consequences for research consumers such as scientists, clinicians, policy makers, the media, and patients [13,14]. Researchers can minimize this source of waste by adhering to reporting guidelines, which define a minimum set of information required when reporting medical research to ensure its usefulness [15]. One of the first reporting guidelines was the Consolidated Standards of Reporting Trials (CONSORT) for writing up reports of RCTs, developed in 1996 and updated in 2001 and 2010. CONSORT consists of a 25-item checklist and flow diagram to help authors report their RCTs [16]. Item 5 of CONSORT 2010 concerns an RCT’s intervention. To improve reporting completeness and replicability of interventions, CONSORT item 5 was extended into a 12-item reporting guideline, “the Template for Intervention Description and Replication” (TIDieR) [17]. Other extensions are also available for specific designs (eg, crossover trials) [18], interventions (eg, nonpharmacologic treatment interventions) [19], and data (eg, harms) [20]. Similar guidance to improve reporting completeness for systematic reviews and meta-analyses—the Preferred Reporting Items for Systematic reviews and Meta-Analyses (PRISMA) Statement—was developed in 2009 and updated in 2020 [21].

Journal endorsement of these reporting guidelines is associated with better reporting [22-24]. For example, a Cochrane review including meta-research studies assessing the reporting completeness of RCTs demonstrated a standardized mean difference of 0.68 (99% CI 0.38 to 0.98) on the aggregate score of all CONSORT items in favor of CONSORT-endorsing journals over nonendorsers. Allocation concealment, scientific rationale in the introduction, sample size, and sequence generation were also more adequately described in RCTs published in CONSORT-endorsing journals [22]. A study evaluating journal instructions for authors found that 27 out of 33 high-impact nutrition and dietetic journals with an impact factor over 2.88 mentioned CONSORT [23]. In a cross-sectional study in the field of nursing, the median rates of adherence to the PRISMA statement were 73% (IQR 59.5%-94.6%) for reviews published in journals that endorsed PRISMA and 64.9% (IQR 17.6%-92.3%) for reviews published in journals that did not endorse PRISMA [24]. As far as we know, the correlation between reporting completeness and journal endorsement of PRISMA has not been explored in the nutrition field.

Few studies assessed reporting completeness of nutrition research. Although previous studies have found poor reporting in nutrition- and diet-related studies [25-31], most focused on the association of particular funding sources with methodological and reporting quality [25-27,30,31] or risk of bias [28,29]. We identified only 1 systematic review of RCTs assessing the quality of reporting in RCTs of dietetic interventions in primary care. The authors found that none of the 27 included RCTs reported all CONSORT items, with a median of 11.5 of the 28 items completely met, 8 partially met, and 10 not met at all. The items most often not reported were “a description of where the full protocol can be accessed (n=26, 96%), ”registry number and name of trial registry“ (n=23, 85%), ”mechanism used to implement the random allocation sequence“ (n=22, 81%), and ”who generated the random allocation sequence, enrolled participants and assigned participants'' (n=22, 81%) [32]. We did not find any study assessing the completeness of reporting of systematic reviews in nutrition. One scoping review evaluated 57 studies that assessed adherence to individual PRISMA items, where 79% (n=45) of them focused on therapeutic interventions and related to surgery clinical areas. It identified 11 items that are fewer than 67% of systematic reviews adhered to, including structured summary, protocol and registration, search, data items, risk of bias in individual and across studies (in both methods and results sections), additional analyses, and funding [11].

CONSORT and TIDieR do not cover all possible practices to enhance research reproducibility, such as the inclusion of data-sharing and code-sharing statements. A survey of a random sample of 149 biomedical articles published between 2015 and 2017 found that 18.3% discussed publicly available data [33]. Similarly, an automated assessment of all PubMed Central articles estimated that in 2020, overall 15% of articles had a data-sharing statement and 3% had a code-sharing statement [34].

“Spin,” as defined by Boutron et al [35], is the use of specific reporting strategies, from whatever motive, to highlight that the experimental treatment is beneficial, despite a statistically nonsignificant difference for the primary outcome, or to distract the reader from statistically nonsignificant results.

Spin is frequently found in the medical literature, regardless of study design, and its presence can affect the interpretation of results. A systematic review of 35 reports which investigated spin found that more than 56% of trials included spin in their abstract (n=13) and main text (n=16) and that 26.3% of systematic reviews (n=2) included spin in their main text [36]. We did not identify any studies assessing reproducible research practices or the presence of spin in nutrition systematic reviews or meta-analyses.

### Objectives

This protocol describes 2 independent meta-research studies that aim to (1) assess the reporting completeness of diet- and nutrition-related RCTs, identify their main reporting limitations, and estimate the frequency of reproducible research practices among them; and (2) assess the reporting completeness of systematic reviews with meta-analysis of diet- and nutrition-related RCTs, identify their main reporting limitations, evaluate their risk of bias, and estimate the frequency of spin among them.

## Methods

### Design and Research Questions

Two meta-research studies will be conducted to answer the research questions presented in Textbox 1. Both protocols are registered in the Open Science Framework (doi: 10.17605/OSF.IO/BF47G and 10.17605/OSF.IO/ZTFYX).

**Research questions.**

**Meta-research study of diet- and nutrition-related randomized controlled trials (meta-research study 1)**

- What is the proportion of items adherent to CONSORT (Consolidated Standards of Reporting Trials) and TIDieR (Template for Intervention Description and Replication) in a sample of published randomized controlled trials of diet- or nutrition-related interventions?
- What is the frequency of reproducible research practices among these randomized controlled trials?

**Meta-research study of systematic reviews with meta-analysis of diet- and nutrition-related randomized controlled trials (meta-research study 2)**

- What is the proportion of items adherent to PRISMA (Preferred Reporting Items for Systematic reviews and Meta-Analyses) in a sample of published systematic reviews with meta-analysis of diet- and nutrition-related randomized controlled trials?
- What is the risk of bias in these reviews?
- What is the frequency of spin in the abstract and the main text of these reviews?

### Eligibility Criteria

For meta-research study 1, we will include diet- and nutrition-related RCTs published in peer-reviewed journals. We will consider self-identification as an RCT as an inclusion criterion. All diet- and nutrition-related RCTs, regardless of the population or outcomes evaluated or the framework design will be included (eg, noninferiority, superiority, or exploratory). There will be an exclusion of non-RCTs, pilot and feasibility trials, studies that assess pharmaceutical or herbal medicines as an intervention, and trials without isolated nutrition- or diet-related interventions (meaning that trials combining more than 1 intervention will not be eligible). RCTs that do not include any term related to the categories of intervention described in Textbox 2 in their titles or abstracts will be excluded.

For meta-research study 2, systematic reviews will be included with meta-analysis of diet- and nutrition-related RCTs published in peer-reviewed journals. All Cochrane publications will be excluded as their moderate to high methodological quality is likely to ensure a high level of reporting completeness [37].

**Categories of nutrition- or diet-related interventions.**

- Food (whole food, food products, specially formulated foods)
- Breastfeeding
- Complete diet or dietary patterns
- Complete nutrition formulas (enteral or parenteral)
- Supplementation or supplements (single or multiple nutrients, bioactive nonnutrients, and plant components)
- Nutrition education, counseling, and coordination of care

We adapted the categories proposed by Naude et al [37] to include “shared foods of complete diet and dietary patterns” and “enteral or parenteral formulas” and remove “nutrition-related policies.”

For both meta-research studies, the language of publications will not be an eligibility criterion aiming to avoid language bias. Our team has researchers proficient in English, Portuguese, and Spanish. If necessary, Google Translate or private translation services will be used to extract data from publications in other languages. The nutrition intervention categories of interest are listed in Textbox 2 [37].

### Literature Research

For meta-research study 1, we will use the search strategy developed by Durão et al [38] to identify RCTs of nutritional interventions [32]. For meta-research study 2, a search strategy is constructed using nutrition- and diet-related terms [38] combined with a validated search strategy to identify systematic reviews or meta-analyses [39]. No limitation will be set for language during the literature search. PubMed search is conducted (via the National Library of Medicine) using the search strategies presented in Multimedia Appendices 1 and 2. There will be a search for RCTs (June 1, 2021, to June 1, 2022) and systematic reviews with meta-analysis (July 01, 2021, to July 01, 2022) indexed in 1 calendar year.

### Study Selection and Sample Size

For both meta-research studies, we will organize the retrieved reports in EndNote (Clarivate). We will remove duplicates using EndNote’s automated deduplication, then manually check for and exclude any remaining duplicates. The final library without duplicates to Rayyan will be imported [40], where 2 reviewers will independently determine the eligibility of each report in a 2-stage process. They will screen titles and abstracts and select all RCTs and systematic reviews with meta-analysis assessing any diet- or nutrition-related intervention. Liberal acceleration will be applied, in which both reviewers must exclude a record for it to be excluded, while only 1 reviewer must include a record for it to be included. The reviewers will then read the full texts to confirm study eligibility. In both screening rounds, any discrepancies will be resolved through discussion between the reviewers (FS and JL, acknowledged), with adjudication by a third reviewer (MSM). All reviewers have experience in conducting systematic reviews.

The eligible RCTs and systematic reviews with meta-analysis will be grouped according to the intervention categories described in Textbox 2, and the proportion of RCTs and systematic reviews in each category is calculated. A convenience sample of 100 RCTs will be randomly selected for inclusion in meta-research study 1 and 100 random systematic reviews with meta-analysis for meta-research study 2 that meet the above criteria, with no formal sample size calculation. We aim to maintain the proportion of each intervention category (Textbox 2) in both meta-research studies, for example, for a given intervention category that comprises, say, 50% of the total sample of potentially eligible RCTs, we will randomly select 50 of those for our sample of 100 RCTs. A web-based random number generator will be used to create the list of publications to include from an enumerated sequence of all studies in each intervention category [41].

A flowchart will be presented to demonstrate each step of the study selection process. A list of the studies excluded at the full-text screening stage will be presented with reasons for their exclusion for both meta-research studies.

### Data Extraction

For meta-research study 1, a total of 2 reviewers will independently extract data from the selected RCTs to evaluate their reporting completeness, identify their main reporting limitations, and assess their use of predefined reproducible research practices. We have developed a data extraction form (Multimedia Appendix 3) that includes (1) publication data, such as PMID, first author’s name, journal in which the RCT was published, and the journal’s field according to Web of Science. (2) Basic description of RCT data, categorized into population, intervention, comparator, outcome, and study design (PICOS) domains, adapted from Naude et al [37]. (3) Use of practices that promote research transparency and reproducibility, such as the use of CRediT [42] to acknowledge authorship; availability of a full protocol publication, statistical analysis plan, and materials/data/code; open access; disclosure of funding source; and disclosure of conflicts of interest. All these practices will be categorized as “yes or no.”

An additional data extraction form to evaluate adherence to items from CONSORT [16], its extensions for abstracts [43], harms [20], crossover [18], nonpharmacological [19], cluster trials [44], and TIDieR [15] statements will be developed. Each item will be evaluated as fully reported, not reported, partially reported, or not applicable. As there is an inclusion of RCTs published after the COVID-19 pandemic began, items from the CONSERVE reporting guideline will also be included. CONSERVE was developed to improve the reporting completeness of trials modified due to the COVID-19 pandemic or other extenuating circumstances [45] (Multimedia Appendix 4).

For meta-research study 2, a total of 2 reviewers will independently extract data from the selected systematic reviews with meta-analysis of nutrition- or diet-related RCTs. We have developed a data extraction form (Multimedia Appendix 5) to capture (1) Publication data, such as PMID, first author’s name, journal in which the meta-analysis was published, and the journal’s field according to Web of Science. (2) Meta-analysis research questions, categorized into PICOS domains, adapted from those used by Naude et al [37]. (3) The presence of spin in the abstract and the main text, classified into four main categories: (A) misleading reporting, including 10 items in the main text and 8 items in the abstract; (B) misleading interpretation, including 13 items in the main text and 10 items in the abstract; (C) inappropriate extrapolation, including 5 items in the main text and 3 items in the abstract, as proposed by Yavchitz et al [46]; and (D) methodological quality according to the ROBIS tool. Phases 2 and 3 of the tool will be followed to make an overall judgment of risk of bias (which will be rated as low, high, or unclear) [47].

An additional data extraction form to evaluate adherence to PRISMA [21], its extensions [48,49], and TIDieR [15] items was also developed (Multimedia Appendix 6).

For both studies, we will pilot-test the data extraction forms on 5 randomly selected full-texts before proceeding with full data extraction to ensure consistency in the interpretation of data items and refine the data extraction form, if necessary. All extracted information will be entered directly into the study databases using REDCap [50]. Any disagreement between the pairs of reviewers will be resolved by a third reviewer.

### Data Analysis

For both meta-research studies, descriptive aspects of included publications (ie, data on bibliometrics, PICOS, research transparency practices, presence of spin, and risk of bias) will be presented both as absolute and relative frequencies, either in diagrams or tables.

For meta-research 1, calculation of an aggregate score of adherence to all CONSORT, its extensions, CONSERVE, and TIDieR items will be presented. The items of relevant reporting guidelines’ checklists in 170 questions with categorical answers (Multimedia Appendix 3) were fragmented. Most questions presented 2 options as answers (yes or no), whereas some questions presented 3 or 4 options as answers (yes, no, partially, or not applicable). Answers “yes” will be scored as 1 point, while answers “no” will be scored as 0 points and answers “partially” will be scored as 0.5 points. Answers such as “not applicable” will be discounted for that particular study. Therefore, the final score can range from 0 to 170.

For meta-research 2, we will similarly calculate an aggregate score of adherence to all PRISMA, its extensions, and TIDieR items. For meta-research 2, all items of the corresponding reporting guidelines were fragmented into 114 questions with the same possible categorical answers (and respective attributable scores) described above (Multimedia Appendix 6). Thus, the final score for the meta-research of meta-analyses can range from 0 to 114.

In the final report, the results will be presented as frequencies (and respective percentages in relation to the total number of relevant studies) for individual items and proportion of the total achievable score for individual studies. No data aggregation or cutoff points are anticipated for the descriptive analyses.

The general features between RCTs and meta-analyses with above-average and below-average reporting score proportions were also compared, which are stratified by the mean or median value (depending on the distribution) using the Student *t* test or Mann-Whitney test for quantitative variables and the chi-square test for categorical variables. Logistic regression models will be constructed to explore the determinants of reporting completeness.

Any changes to the protocol will be detailed and justified in the publications describing the results of the study.

### Ethical Considerations

As both meta-research studies will involve reviewing and collecting evidence from previously published papers, ethical approval is not required. Both meta-research studies will be reported according to the PRISMA adaptation for meta-epidemiological methodology research [51]. Any updates and amendments to this protocol will be described and justified in the final publications. The results will be published in peer-reviewed journals.

## Results

The literature search for the first meta-research study retrieved 20,030 records and 2182 were potentially eligible. Among them, 100 nutrition- or diet-related RCTs were randomly selected for data extraction.

The literature search for the second meta-research study retrieved 10,918 records and 850 were potentially eligible. Among them, a random sample of 100 systematic reviews with meta-analysis was selected for data extraction.

Data extraction of both meta-research studies is currently in progress and the completion is expected by the beginning of 2023.

## Discussion

We presented the protocols of 2 independent meta-research studies aiming to investigate the completeness of reporting and the frequency of research transparency practices in a random sample of nutrition- or diet-related RCTs and meta-analyses published in journals indexed in PubMed.

Nutrition interventions have particular challenges that need careful consideration during all study phases and detailed communication of research questions and findings. Thus, well-written and complete reporting of RCTs and meta-analyses of RCTs are crucial to support scientific integrity, ethical standards, safety, and validation of study methods and findings [4]. Inappropriate reporting of research frequently means an important and avoidable source of research waste [12], with consequences for scientists, clinicians, policy makers, the media, and patients [13,14]. Researchers can minimize this waste by adhering to CONSORT [16], TIDieR [17], and PRISMA [21] when reporting their work. Indeed, additional practices to enhance research reproducibility such as the adoption of reproducible workflows and open science practices, including data and code-sharing, can benefit nutrition- and diet-related research [52].

The complexity of reporting intervention studies depends on the type of intervention under investigation, as well as knowledge of relevant extensions of general reporting guidelines for specific fields. Nutrition- or diet-related interventions have several particularities, and a detailed evaluation of whether RCTs and meta-analyses of nutritional interventions adhere to CONSORT [16] and PRISMA [21] statements, respectively, is essential. As per our knowledge, this will be the first meta-research study addressing the main reporting limitations of nutrition- or diet-related intervention studies.

At 1-year interval, we identified 2182 and 850 publications describing potentially eligible RCTs and systematic reviews with meta-analysis, respectively. These numbers illustrate the relevance of these types of publications in the nutrition research field, as well as the need for a detailed evaluation of their reporting completeness and transparency. The fact that our samples do not represent all publications in this field has been acknowledged as a study limitation. However, our search strategy choices are based on the premise that the eligible publications retrieved should provide enough evidence to fulfill our aims, as they reflect a recent body of scientific literature indexed in the main database for medical research. While not including Cochrane reviews is also a limitation of our research, their inclusion could bias our findings since these publications follow specific guidelines (eg, for content, structure, and format), and have overall higher methodological quality than standard peer-reviewed publications. Future research could explore the main differences between Cochrane and non-Cochrane systematic reviews of nutritional- or diet-related interventions.

These meta-researchers will inform further deliberations around the need to develop specific reporting guidelines for nutrition- and diet-related RCTs and systematic reviews with meta-analysis since this evaluation can shed light on the main reporting limitations in the area. Our meta-research studies will fill this gap. Therefore, we aim to help increase in the transparency and reproducibility of studies involving nutrition- or diet-related interventions.

## Appendix

Multimedia Appendix 1 Full search strategy for PubMed database (meta-research study 1—nutrition- and diet-related RCTs).

Multimedia Appendix 2 Full search strategy for PubMed database (Meta-research study 2—systematic reviews with meta-analyses of nutrition- and diet-related RCTs).

Multimedia Appendix 3 Standardized form for data extraction for diet- or nutrition-related RCTs published as scientific articles in peer-reviewed journals.

Multimedia Appendix 4 Data extraction form: completeness reporting of a recent sample of nutrition- or diet-related RCTs published in peer-reviewed journals.

Multimedia Appendix 5 Standardized form for data extraction for systematic reviews with meta-analyses of diet- or nutrition-related RCTs published as scientific articles in peer-reviewed journals.

Multimedia Appendix 6 Data extraction form: completeness reporting of a recent sample of meta-analyses of nutrition or diet-related RCTs published in peer-reviewed journals.

## Abbreviations

CONSORT: Consolidated Standards of Reporting Trials
RCT: randomized controlled trial
TIDieR: Template for Intervention Description and Replication
PICOS: population, intervention, comparator, outcome, and study design
PRISMA: Preferred Reporting Items for Systematic reviews and Meta-Analysis

## Data Availability

Data will be maintained in .csv, .txt, .ris, .docx, and .pdf format for storage for the period of the study and beyond. Extracted data will be deposited in digital open access repositories, such as UK Data Service or Oxford Research Archive.
