# Supplementary material for: Completeness of Reporting in Diet- and Nutrition-Related Randomized Controlled Trials and Systematic Reviews With Meta-Analysis: Protocol for 2 Independent Meta-Research Studies
Source: JMIR Res Protoc. 2023 Mar 23;12:e43537. doi: 10.2196/43537 (PMC10131600; doi:10.2196/43537)
Supplement: Multimedia Appendix 2 [file resprot_v12i1e43537_app2.docx]

**Multimedia Appendix 2. Full search strategy for PubMed database (Meta-research study 2—systematic reviews with meta-analyses of nutrition- and diet-related RCTs).**

Search filter: Modified McMaster Hedges Maximizes specificity filter

https://hiru.mcmaster.ca/hiru/HIRU_Hedges_MEDLINE_Strategies.aspx#Reviews

#1 (Nutritional Sciences[mh] OR Nutritional Physiological Phenomena[mh] OR Nutrition Assessment[mh] OR Nutrition Therapy[mh] OR Nutritional and Metabolic Diseases[mh] OR nutrition*[tiab] OR diet[tiab] OR feeding[tiab] OR dietary[tiab] OR breastfeed*[tiab] OR breast feed*[tiab] OR lactation[tiab] OR bottle feed*[tiab] OR complementary feeding[tiab] OR weaning[tiab] OR enteral[tiab] OR parenteral[tiab] OR Feeding Methods[mh] OR nutritional status[tiab] OR overweight[tiab] OR obese[tiab] OR obesity[tiab] OR overnutrition[tiab] OR over nutrition[tiab] OR undernourished[tiab] OR overnourished[tiab] OR wasted[tiab] OR wasting[tiab] OR stunting[tiab] OR stunted[tiab] OR underweight[tiab] OR undernutrition[tiab] OR under nutrition[tiab] OR body weight[tiab] OR anthropometry[tiab] OR Body Weights and Measures[mh] OR growth monitoring[tiab] OR food[tiab] OR food labelling[mh] OR food assistance[mh] OR supplementary feeding[tiab] OR diet therapy[mh] OR food and beverages[mh] OR vegetable*[tiab] OR fruit*[tiab] OR meat[tiab] OR dairy[tiab] OR dietary fat*[tiab] OR starch*[tiab] OR cereal[tiab] OR food-drug interactions[mh] OR food supply[mh] OR feeding behavio*[tiab] OR eating behavio*[tiab] OR food pattern*[tiab] OR food hypersensitivity[mh] OR food deprivation[mh] OR food, organic [mh] OR micronutrient*[tiab] OR vitamin*[tiab] OR thiamin[tiab] OR riboflavin[tiab] OR niacin[tiab] OR pantothenic acid[tiab] OR pyridoxine[tiab] OR pyridoxal[tiab] OR pyridoxamine[tiab] OR biotin[tiab] OR folic acid[tiab] OR folate[tiab]OR cyanocobalamin[tiab] OR choline[tiab] OR retinol[tiab] OR ascorbic acid[tiab] OR tocopherol[tiab] OR carotenoids[tiab] OR carotene[tiab] OR cryptoxanthin[tiab] OR lutein[tiab] OR lycopene[tiab] OR zeaxanthin[tiab] OR minerals[tiab] OR calcium[tiab] OR chloride[tiab] OR magnesium[tiab] OR phosphorus[tiab] OR potassium[tiab] OR sodium[tiab] OR iron[tiab] OR sulphur[tiab] OR trace element*[tiab] OR boron[tiab] OR cobalt[tiab] OR chromium[tiab] OR copper[tiab] OR fluoride[tiab] OR iodine[tiab] OR iron[tiab] OR manganese[tiab] OR molybdenum[tiab] OR selenium[tiab] OR zinc[tiab] OR trace metal*[tiab] OR macronutrient*[tiab] OR carbohydrate*[tiab] OR dietary protein*[tiab] OR saturated fat*[tiab] OR unsaturated fat*[tiab] OR mono unsaturated fat*[tiab] OR monounsaturated fat*[tiab] OR poly unsaturated fat*[tiab] OR polyunsaturated fat*[tiab] OR trans fat*[tiab] OR dietary fibre[tiab] OR dietary fiber[tiab] OR dietary salt[tiab] OR table salt[tiab] OR soft drink[tiab] OR fruit juice[tiab] OR vegetable juice[tiab] OR milk[tiab] OR tea[tiab] OR coffee[tiab] OR energy drink*[tiab] OR carbonated beverage*[tiab] OR carbonated drink*[tiab] OR prebiotics[tiab] OR probiotics[tiab] OR glycemic load[tiab] OR glycemic index[tiab] OR glycaemic load[tiab] OR glycaemic index[tiab] OR calories[tiab] OR kilocalories[tiab] OR kilojoules[tiab] OR caloric intake[tiab] OR energy intake[tiab])

#2 meta-analysis [pt] OR systematic review [pt]

#3 MEDLINE [tiab] OR (systematic [tiab] AND review [tiab])

#4 #2 OR #3

#5 2021/07/01:2022/07/01[Date - Publication]

#6 #1 AND #4 AND #5
