# Supplementary material for: Completeness of Reporting in Diet- and Nutrition-Related Randomized Controlled Trials and Systematic Reviews With Meta-Analysis: Protocol for 2 Independent Meta-Research Studies
Source: JMIR Res Protoc. 2023 Mar 23;12:e43537. doi: 10.2196/43537 (PMC10131600; doi:10.2196/43537)
Supplement: Multimedia Appendix 3 [file resprot_v12i1e43537_app3.docx]

**Multimedia Appendix 3. Standardized form for data extraction for diet- or nutrition-related RCTs published as scientific articles in peer-reviewed journals.**

*Publication and RCT features*

1. Study ID: ______

2. First author:_________________

3. Number of authors: _________

4. Journal: __________________

5. PMID/ DOI: ________________________________

6. PICOS:

6.1.1. Participants:

( ) Pregnant women ( ) Mother and infant pairs

( ) Infants ( ) Children and preschool-aged children

( ) Adults ( ) Elderly

( ) Adults and elderly ( ) Postmenopausal women

( ) Participants with a clinical condition (s) (specify it)____________

6.1.2. Participants:

Cancer ( ) yes ( ) no

Cardiovascular disease ( ) yes ( ) no

6.2. Intervention:

( ) Food (whole food, food products, specially formulated foods)

( ) Breastfeeding

( ) Complete diet or dietary patterns

( ) Complete nutrition formulas (enteral or parenteral)

( ) Supplementation or supplements (single or multiple nutrients, bioactive non-nutrients, plant components)

( ) Nutrition education, counseling and coordination of care

( ) Other, if no component of intervention could be categorized as any of the above (specify it)____________

6.3. Comparator:

( ) Placebo

( ) No intervention

( ) Usual care

( ) Different intervention

( ) Other (specify it)____________

6.4. Primary outcome:

( ) Not specified

( ) Mortality

( ) Clinical status (clinical or biochemical measures)

( ) Nutritional status (anthropometry, body composition, nutrition diagnosis)

( ) Frequency or severity of disease

( ) Diet quality and/or variety

( ) Food/ nutrient/ dietary intake

( ) Diet-related behaviors

( ) Other non-dietary behaviors

( ) Withdrawal from the study, drop-out or adherence-related

( ) Adverse events, side-effects and/or safety

( ) Cost-effectiveness or economic

( ) Quality of life

( ) Other (specify it)____________

6.5.1 Study design:

( ) parallel RCT ( ) crossover RCT

( ) cluster RCT ( ) non-cluster RCT

( ) factorial ( ) non-factorial

( ) two arms ( ) multi-arms

( ) unicentric ( ) multicentric

6.5.3. Study Framework:

( ) superiority

( ) inferiority

( ) equivalence

( ) exploratory

( ) not specified

*Reproducibility and transparency*

7. If funding received, specify the funder:

( ) National Institute Health

( ) Other federal agency

( ) Industry

( ) Individuals

( ) Universities

( ) Organizations

( ) Other

8. Pre-registration statement

( ) Statement declaring study was pre-registered

( ) Statement declaring the study was not pre-registered

( ) No pre-registration statement present

9. Full statistical analysis plan statement

( ) Statement declaring that the statistical analysis plan is available

( ) Statement declaring that the statistical analysis plan is not available

( ) No statistical analysis plan statement present

10. If a statement declaring that the statistical analysis plan is available, where it can be accessed:

( ) It was published as a preprint

( ) It was published as a peer-reviewed article

( ) Other. Specify: __________________

11. Other material availability:

( ) Statement declaring that other materials are available

( ) Statement declaring that materials are not available

( ) No materials availability statement present

12. Data sharing:

( ) Statement declaring that the data (or some of the data) are publicly available

( ) Statement declaring that the data are not publicly available

( ) Statement declaring that data are available upon request.

( ) No data availability statement present

13. Code sharing:

( ) Statement declaring that code is publicly available

( ) Statement declaring that code is not publicly available

( ) Statement declaring that code is available upon request

( ) No code availability statement present

14. Conflict of interest statement

( ) Disclosure statement present, authors declare one or more conflicts of interest

( ) Disclosure statement present, authors declare there are no conflicts of interest

( ) No conflict of interest statement present

15. Open access

( ) Open Access publication

( ) No open access publication

16. Use of CRediT (https://casrai.org/credit/) to acknowledge authorship

( ) no ( ) yes

Yes if the following items are described (not necessary all them): Conceptualization, Data curation, Formal Analysis, Funding acquisition, Investigation, Methodology, Project administration, Resources, Software, Supervision, Validation, Visualization, Writing – original draft, Writing – review & editing
