# Supplementary material for: Completeness of Reporting in Diet- and Nutrition-Related Randomized Controlled Trials and Systematic Reviews With Meta-Analysis: Protocol for 2 Independent Meta-Research Studies
Source: JMIR Res Protoc. 2023 Mar 23;12:e43537. doi: 10.2196/43537 (PMC10131600; doi:10.2196/43537)
Supplement: Multimedia Appendix 4 [file resprot_v12i1e43537_app4.docx]

**Multimedia Appendix 4. Data extraction form: completeness reporting of a recent sample of nutrition- or diet-related RCTs published in peer-reviewed journals.**

|  | | **Item** | **Description** | | **Reported** |
| --- | --- | --- | --- | --- | --- |
| **TITLE AND ABSTRACT** | | | | | |
| 1 | | Consort_1a | **Title** - Is the study design described in the title? (i.e. is it identified as a randomised trial and specify if the study is a parallel, crossover, cluster, factorial, etc,?) | | [ ] No  [ ] Yes |
| 2 | | Consort_1b | **Abstract** - Is there a structured summary of trial design, methods, results and conclusions? | | [ ] No  [ ] Yes |
| **INTRODUCTION** | | | | | |
| 3 | Consort_2a | | | **Scientific Background and rationale** - Is there an explanation of the scientific background and rationale of the trial? [rationale may be explanatory (e.g. to assess the possible influence of a drug on renal function) or pragmatic (e.g. to guide the practice by comparing the benefits and harms of different treatments)]. | [ ] No  [ ] Yes |
| 4 | Consort_2a  Consort_H2 | | | **Scientific Background and rationale** - Is presented a summary of relevant studies (published or unpublished) examining benefits and harms for the main intervention? (Partially, if presented studies examining benefits or harms, but not both) | [ ] No  [ ] Partially  [ ] Yes |
| 5 | Consort_2a  Consort_H2 | | | **Scientific Background and rationale** - Is presented a summary of relevant studies (published or unpublished) examining benefits and harms for the other interventions? (if placebo or no intervention, N/A. Partially, if presented studies examining benefits or harms, but not both) | [ ] No  [ ] Partially  [ ] Yes  [ ] N/A |
| 6 | Consort_2b | | | **Objectives** - Are the specific objectives described? | [ ] No  [ ] Yes |
| 7 | Consort_2b | | | **Objectives** - Are the specific hypotheses described? | [ ] No  [ ] Yes |
| 8 | Consort_Cluster_2b | | | **Objectives (cluster trials)** - Is it clear whether objectives and hypotheses pertain to the individual participant level, the cluster level, or both? | [ ] No  [ ] Yes  [ ] N/A |
| 9 | Consort_SPI_2b | | | **Objectives or hypotheses (SPI trials)** - if pre-specified, is it described how the intervention was hypothesised to work? (authors should describe the 'mechanism of action', also known as 'theory of challenge', 'programme theory' or 'causal pathway'. Graphical depictions may be accepted as a description)  N/A when the intervention is not related to nutrition education or counselling. | [ ] No  [ ] Yes  [ ] N/A |
| **METHODS** | | | | | |
| 10 | | Consort_3a | | **Trial design** - Is there a description of trial design, including type of trial (e.g., parallel group, crossover, factorial, cluster, single group)? | [ ] No  [ ] Yes |
| 11 | | Consort_3a | | **Trial design** - Is there a description of trial design, including allocation ratio (e.g. 1:1 or 2:1, in two-arms parallel trials)?  N/A if a crossover design. | [ ] No  [ ] Yes  [ ] N/A |
| 12 | | Consort_3a | | **Trial design** - Is there a description of trial design, including framework (e.g., superiority, equivalence, non-inferiority, exploratory)? (it can be identified by the hypothesis when it is described). | [ ] No  [ ] Yes |
| 13 | | Consort_3a  Consort_3a_Crossover | | **Trial design** - If a less common design is employed, did the authors explain their choice? (e.g. factorial, cluster, multi-arm, crossover) | [ ] No  [ ] Yes |
| 14 | | Consort_3a_Crossover | | **Trial design (crossover trials)** - Is it described the number and duration of periods?  N/A if not a crossover design. | [ ] No  [ ] Yes  [ ] N/A |
| 15 | | Consort_3a_Crossover | | **Trial design (crossover trials)** - Is it described the duration of washout period and consideration of carry over effect?  N/A if not a crossover design. | [ ] No  [ ] Yes  [ ] N/A |
| 16 | | Consort_Cluster_3a | | **Trial design (cluster trials)** - Is it described a definition of clusters?  (Cluster is the unit of randomisation and it should be appropriately defined)  N/A if not a cluster trial.. | [ ] No  [ ] Yes  [ ] N/A |
| 17 | | Consort_Cluster_3a | | **Trial design (cluster trials)** - Is it described how design features apply to clusters?  (e.g. whether the cluster randomised design is parallel, matched pair, or other, and whether the treatments have a factorial structure)  N/A if not a cluster trial.. | [ ] No  [ ] Yes  [ ] N/A |
| 18 | | Consort_NPT_3a  Consort_SPI_5c | | **Trial design -** When applicable, is there a description of how care providers were allocated to each trial group?  It should be described if the same team of researchers performed both interventions or how it was defined the allocation of them to each trial group, and the number of participants assigned to each provider.  (N/A if the intervention is not related to nutrition education or counselling) | [ ] No  [ ] Yes  [ ] N/A |
| 19 | | Consort_3b | | **Important changes to methods after trial commencement, with reasons** - Is there a description of any change due to external information becoming available from other studies, or internal financial difficulties, or a disappointing recruitment rate? | [ ] No  [ ] Yes |
| 20 | | Consort_3b  CONSERVE_4 | | **Important changes to methods after trial commencement** - is there a clear description that protocols changes were made without breaking the blinding on the accumulating data on participants' outcomes?  (N/A if an open label study) | [ ] No  [ ] Yes  [ ] N/A |
| 21 | | CONSERVE_1 | | **Extenuating Circumstances** - Is it described any extenuating circumstance that lead to the trial modifications, including how the circumstances are extenuating?  * *Extenuating circumstances refer to unavoidable situations that prompt modifications to a trial and that are not usually under the control of study investigators, sponsors, or funders (e.g. COVID pandemic, wars, strikes, riots, or epidemics that prevent parties from fulfilling their contractual obligations)* | [ ] No  [ ] Yes |
| 22 | | CONSERVE_2a | | **Important Modifications -** Is it described how the modifications are important modifications?  * Important modi*fications are these that could have a potentially meaningful effect on the study objectives or research question, ethical acceptability (including benefits and harms to participants), internal validity, generalizability, feasibility, or analytical methods and statistical power)*  *(N/A if 35 is no)* | [ ] No  [ ] Yes  [ ] N/A |
| 23 | | CONSERVE_2b | | **Important Modifications -** Is it described the impacts of the important modifications, including their rationale and implications for the trial? ** Impacts refer to aspects of the trial that are directly affected or changed by the extenuating circumstance and that are not under the control of investigators, sponsors, or funders.*  *(N/A if 35 is no)* | [ ] No  [ ] Yes  [ ] N/A |
| 24 | | CONSERVE_2c | | **Important Modifications -** Is it described the mitigating strategies, including their rationale and implications for the trial?  * *Mitigating strategies refer to the aspects of the trial that are modified by the study investigators, sponsor, or funder in response to the extenuating circumstances or to manage the impacts on the trial. (N/A if 35 is no)* | [ ] No  [ ] Yes  [ ] N/A |
| 25 | | CONSERVE_2d | | **Important Modifications -** Is it provided a modifications timeline?  N/A if no modification is mentioned. | [ ] No  [ ] Yes  [ ] N/A |
| 26 | | CONSERVE_3 | | **Responsible Parties -** Is it stated who planned, reviewed and approved the modifications?  N/A if no modification is mentioned. | [ ] No  [ ] Yes  [ ] N/A |
| 27 | | Consort_4a | | **Eligibility criteria** - Are the inclusion and exclusion criteria for participants described? | [ ] No  [ ] Yes |
| 28 | | Consort_Cluster_4a | | **Eligibility criteria -** If cluster trials, are the eligibility criteria for study centres provided?  N/A if it is not a cluster trial. | [ ] No  [ ] Yes  [ ] N/A |
| 29 | | Consort_4a | | **Eligibility criteria** - Is there a description of the recruitment method, such as by referral or self-selection (e.g. through advertisements) | [ ] No  [ ] Yes |
| 30 | | Consort_NPT_4a  Consort_SPI_4a | | **Eligibility criteria** **-** When applicable, is there a description of the eligibility criteria for centres (settings) and for care providers?  (N/A if the intervention is not related to nutrition education or counselling) | [ ] No  [ ] Yes  [ ] N/A |
| 31 | | Consort_4b | | **Study setting - Environment:** Is there a detailed description of study settings, including country, city if applicable, and immediate environment? (e.g., community, office practice, hospital clinic, or inpatient unit)  Partially if it is not presented a detailed description of study setting and No if no information is described. | [ ] No  [ ] Partially  [ ] Yes |
| 32 | | Consort_4b | | **Study setting - Structure:** Is there a detailed description of study settings, with a clear description whether the trial was carried out in one (single-centre) or several centres (multicentre trials)? | [ ] No  [ ] Yes |
| 33 | | Consort_4b | | **Study setting - Location:** Is it presented a list of study centres and countries where data will be collected if it was a multicentre trial?  N/A if it is not a multicentre trial. | [ ] No  [ ] Yes  [ ] N/A |
| Consort_5 | | | | *Covered by the items of TIDieR, 1-12, as outlined below.* | |
| 34 | | TIDieR_1 | | **Interventions description:** Is it provided the name or a phrase that describes the intervention (s)? | [ ] No  [ ] Yes |
| 35 | | TIDieR_2 | | **Interventions rationale:** Is it described any rationale, theory, or goal of the elements essential to the intervention (s)? | [ ] No  [ ] Yes |
| 36 | | TIDieR_3  Consort_ SPI_5b | | **Intervention materials:** Is it described any physical or informational materials used in the intervention(s), including those provided to participants or used in intervention(s) delivery or in training of intervention (s) providers? It is provided information on where the materials can be accessed (e.g. online appendix, URL)?  (‘Yes’ if all criteria fulfilled; ‘Partially’ if where the materials can be accessed not described; ‘No’ if no information on materials was provided.) | [ ] No  [ ] Partially  [ ] Yes |
| 37 | | Consort_Cluster_5 | | **Intervention description (cluster trials);** Is it described whether interventions pertain to cluster level, individual participant level, or both?  N/A if it is not a cluster trial. | [ ] No  [ ] Yes  [ ] N/A |
| 38 | | Consort_NPT_5a | | **Intervention components:** Are the different components of the intervention(s) described?  N/A if the intervention is composed by a unique component (e.g. isolated supplement; an specific diet such as Low-carb diet). | [ ] No  [ ] Yes  [ ] N/A |
| 39 | | Consort_NPT_5b | | **Intervention standardisation:** Is it described whether and how the intervention(s) were standardised?  In cases of interventions such as supplementation, the standardised orientation given to participants should be described. | [ ] No  [ ] Yes |
| 40 | | TIDieR_4 | | **Intervention procedures:** Is it described each of the procedures; activities, and/or processes used in the intervention, including any enabling or support activities?  (‘Yes’ if all criteria fulfilled; ‘Partially’ if any enabling or support activities are not described; ‘No’ if no information on procedures is provided.) | [ ] No  [ ] Partially  [ ] Yes |
| 41 | | TIDieR_5 | | **Interventions providers’ description:** Is it described, for each category of intervention, who was the intervention(s) provider(s) (e.g. psychologist, nursing assistant)?  (‘Yes’ if all criteria fulfilled; ‘Partially’ if described for some interventions categories, but not for all; ‘No’ if no information on intervention providers is given) | [ ] No  [ ] Partially  [ ] Yes |
| 42 | | TIDieR_5 | | **Interventions providers’ background:** Is it described, for each category of intervention provider, their expertise, background and any specific training given?  (‘Yes’ if all criteria fulfilled; ‘Partially’ if described for some interventions categories, but not for all; ‘No’ if no information on intervention providers background is given) | [ ] No  [ ] Partially  [ ] Yes |
| 43 | | TIDieR_6 | | **Interventions delivery modes:** Are the modes of delivery (e.g. face-to-face or by some other mechanism, such as internet or telephone) of the intervention(s) described?  (For complex interventions, mark ‘Yes’ only if delivery modes are described for all intervention components) | [ ] No  [ ] Partially  [ ] Yes |
| 44 | | TIDieR_6 | | **Interventions delivery structure**: Is it described whether the intervention(s) was provided individually or in a group?  (For complex interventions, mark ‘Yes’ only if the delivery structure is described for all intervention components) | [ ] No  [ ] Partially  [ ] Yes |
| 45 | | TIDieR_7 | | **Interventions delivery environment**: Is it described the type(s) of location(s) where the intervention occurred, including any necessary infrastructure or relevant features?.  (‘Yes’ if all criteria fulfilled; ‘Partially’ if any location aspect is not described; ‘No’ if no information on procedures is provided.) | [ ] No  [ ] Partially  [ ] Yes |
| 46 | | TIDieR_8 | | **Interventions quantity:** Is it described the number of times (e.g. one time or 5 sessions) the intervention(s) was delivered?  (For complex interventions, mark ‘Yes’ only if the intervention schedule is completely described for all intervention components) | [ ] No  [ ] Partially  [ ] Yes |
| 47 | | TIDieR_8 | | **Interventions frequency:** Is it described the frequency (e.g. every two weeks or daily) with which the intervention(s) was delivered.  (For complex interventions, mark ‘Yes’ only if the intervention schedule is completely described for all intervention components) | [ ] No  [ ] Partially  [ ] Yes |
| 48 | | TIDieR_8 | | **Interventions period:** Is it described the period (e.g. 6 months) over which the intervention(s) was delivered? In crossover trials, this must include information about any run-in and washout periods, or a statement on why these are not needed.  (For complex interventions, mark ‘Yes’ only if the intervention schedule is completely described for all intervention components) | [ ] No  [ ] Partially  [ ] Yes |
| 49 | | TIDieR_8 | | **Interventions amount:** Is it described the amount (length of session, or dose) of the intervention that was delivered at each time?  (For complex interventions, mark ‘Yes’ only if the intervention amount is described for all intervention components) | [ ] No  [ ] Partially  [ ] Yes |
| 50 | | TIDIeR_9 | | **Interventions - Tailoring:** Is it described what, why, when, and how the intervention was personalised, titrated or adapted, if applicable? (Only ‘Yes’ if all criteria fulfilled; "Partially" if any criteria of tailoring described; N/A if the intervention is not tailored) | [ ] No  [ ] Partially  [ ] Yes  [ ] N/A |
| 51 | | TIDIeR_10 | | **Interventions modifications-** Is it described if the intervention was modified during the course of the study; describe the changes (what, why, when, and how)? (Only ‘Yes’ if all criteria fulfilled; "Partially" if any criteria of modification described; N/A if the intervention is not modified) | [ ] No  [ ] Partially  [ ] Yes  [ ] N/A |
| 52 | | TIDieR_11 | | **Interventions adherence (planned)** - Are the strategies adopted to improve adherence to intervention protocols (e.g., frequent phone contact) described?  (If a statement explaining that adherence is not relevant to the trial, then ‘Yes’) | [ ] No  [ ] Yes |
| 53 | | TIDieR_11 | | **Interventions adherence (planned)** - Is it described how and by whom the intervention adherence was assessed? (e.g., drug tablet return, laboratory tests)?  (Only ‘Yes’ if all criteria fulfilled or if a statement explaining that adherence is not relevant to the trial; 'Partially' if any criteria described) | [ ] No  [ ] Yes  [ ] Partially |
| 54 | | TIDieR_12  Consort_SPI _5a | | **Interventions adherence (actual)** - Is it described the extent to which the intervention was delivered as planned?  Partially if it is not described for all groups. | [ ] No  [ ] Yes  [ ] Partially |
| 55 | | Consort_NPT_5c | | **Interventions adherence:** Is it described in detail whether and how adherence of care providers to the protocols were assessed or enhanced?  N/A if the intervention is not related to nutrition education or counselling. | [ ] No  [ ] Yes  [ ] N/A |
| 56 | | Consort_6a | | **Outcomes definition** - Is there a clear description of the primary outcome separately than secondary outcomes? ('Yes', if discriminate primary and secondary outcome or if clearly declared that the trial had no secondary outcome) | [ ] No  [ ] Yes |
| 57 | | Consort_Cluster_6a | | **Outcomes description (cluster trials);** Is it described whether outcomes measures pertain to cluster level, individual participant level, or both?  N/A if it is not a cluster trial. | [ ] No  [ ] Yes  [ ] N/A |
| 58 | | Consort_6a | | **Outcomes methods of assessment** - Is there a clear description of the method adopted for assessing the primary outcome(s), if specified, including the specific method(s) of measurement? | [ ] No  [ ] Yes |
| 59 | | Consort_6a | | **Outcomes methods of assessment quality -** Is it reported the use of previously developed and validated scales or consensus guidelines to enhance quality of measurement of the primary outcome(s), if specified? | [ ] No  [ ] Yes |
| 60 | | Consort_6a | | **Outcomes endpoints** - Is it described for the primary outcome(s), if specified, the time of endpoint? | [ ] No  [ ] Yes |
| 61 | | Consort_6a | | **Outcomes assessors** - Is it described for the primary outcome(s), if specified, who was the assessor(e.g. if special skills are required to do so) and how many assessors were used?  Partially if any information was not provided. | [ ] No  [ ] Yes  [ ] Partially |
| 62 | | Consort_6b | | **Any changes to trial outcomes after the trial commenced, with reasons** - Is it presented a detailed reporting of any changes after the trial began of the designation of outcomes as primary or secondary with explanation of why?  ('Yes', if all criteria are fulfilled or a sentence reporting that no changes to trial outcomes was made). | [ ] No  [ ] Yes  [ ] Partially |
| 63 | | Consort_7a | | **Sample size** - Is there a description of how sample size was determined, including (1) the estimated outcomes in each group? | [ ] No  [ ] Yes |
| 64 | | Consort_7a | | **Sample size** - Is there a description of how sample size was determined, including (2) the type I error (alpha)? | [ ] No  [ ] Yes |
| 65 | | Consort_7a | | **Sample size** - Is there a description of how sample size was determined, including (3) the statistical power (type II error or ß)? | [ ] No  [ ] Yes |
| 66 | | Consort_7a | | **Sample size** - Is there a description of how sample size was determined, including (4) for continuous outcomes, the standard deviation of the measurements? (N/A if it was not determined for continuous outcomes) | [ ] No  [ ] Yes  [ ] N/A |
| 67 | | Consort_7a | | **Sample size** - Is there a description of how sample size was determined, including the resulting target sample size per study group? | [ ] No  [ ] Yes |
| 68 | | Consort_7a | | **Sample size** - Is there a description of how sample size was determined, including details of any allowance made for attrition during the study? | [ ] No  [ ] Yes |
| 69 | | Consort_7a | | **Sample size** - Is there a description of how sample size was determined, including details of any allowance made for non-adherence during the study? | [ ] No  [ ] Yes |
| 70 | | Consort_NPT_7a | | **Sample size** - Is there a description of whether and how clustering by care providers or centres was addressed, when applicable?  N/A when clustering by care providers or centres was not relevant. | [ ] No  [ ] Yes  [ ] N/A |
| 71 | | Consort_Cluster_7a | | **Sample size (cluster trials);** Is it described method of calculation, number of clusters, cluster size (s) (and whether equal or unequal cluster sizes are assumed), a coefficient of intracluster correlation (ICC or *k*), and an indication of its uncertainty?  N/A if it is not a cluster trial. | [ ] No  [ ] Yes  [ ] N/A |
| 72 | | Consort_Crossover_7a | | **Sample size (crossover trials)** - Is there a description that an appropriate estimate of within participant variability was used? If a continuous outcome, it is expected standard deviation of the within participant differences be incorporated into the sample size estimation.  (N/A if it is not a crossover trial) | [ ] No  [ ] Yes  [ ] N/A |
| 73 | | Consort_7a | | **Sample size -** Is there a description of why the actual sample size differed from the originally intended sample size (e.g. an interim analyses, poor recruitment or revision of the target sample size) ?  (N/A if sample size intended is equal to the actual sample size) | [ ] No  [ ] Yes  [ ] N/A |
| 74 | | Consort_7a | | **Sample size -** Is there a post hoc calculation of statistical power using the results of the trial instead of a sample size calculation?  ('N/A' if a sample size calculation was presented and the number of patients recruited was in accordance). | [ ] No  [ ] Yes  [ ] N/A |
| 75 | | Consort_7b | | **Interim analyses and stopping guidelines** - when applicable, were these analyses addressed by an independent data monitoring committee or by the authors?  (N/A if no interim analysis was performed). | [ ] No  [ ] Yes  [ ] N/A |
| 76 | | Consort_7b | | **Interim analyses and stopping guidelines** - when applicable, how many interim analyses were conducted?  (N/A if no interim analysis was performed). | [ ] No  [ ] Yes  [ ] N/A |
| 77 | | Consort_7b | | **Interim analyses and stopping guidelines** - when applicable, what triggered them?  (N/A if no interim analysis was performed). | [ ] No  [ ] Yes  [ ] N/A |
| 78 | | Consort_7b | | **Interim analyses and stopping guidelines** - when applicable, is there a description of what statistical methods were used and if they were planned before the start of the trial, before the data monitoring committee saw any interim data by allocation, or some time thereafter?  (N/A if no interim analysis was performed, Partially if they described the statistical methods, but did not report if it was previously planned). | [ ] No  [ ] Yes  [ ] Partially  [ ] N/A |
| 79 | | Consort_8a | | **Randomisation: sequence generation** - Is the method of generating the random allocation sequence described (e.g., computer-generated random numbers)?  Method for generating the allocation sequence refers to the randomisation list (which can be computer-generated or not), rather than to the randomisation method/system itself (which can be computer-based). | [   ] No  [   ] Yes |
| 80 | | Consort_8b | | **Randomisation: type** - Is there a description of whether restriction was used in the randomisation (e.g. simple randomisation was done)? | [   ] No  [   ] Yes |
| 81 | | Consort_8b | | **Randomisation: details of any restriction** - when applicable, is there a description of the methods used to restrict the randomisation? (For block randomisation, authors should provide details on how the blocks were generated, such as by using a permuted block, the block size, and if it was fixed or randomly varied).  N/A if no mention is made related to any restriction on the randomization process. | [   ] No  [   ] Yes  [ ] N/A |
| 82 | | Consort_8b  Consort_Cluster_8b | | **Randomisation: details of stratification** - Is there a specification whether stratification was used, and if so, which factors were involved (e.g. recruitment site, sex, disease stage), the categorisation cut-off values within strata, and the method used for restriction.  (Partially if it is performed a stratified randomization, but it is not specified for which factors). | [   ] No  [   ] Yes  [ ] Partially |
| 83 | | Consort_9 | | **Allocation concealment** - Is the mechanism of implementing the allocation sequence described (e.g., central telephone; sequentially numbered, opaque, sealed envelopes), including any steps to conceal the sequence until interventions are assigned?  Differently from blinding, allocation concealment can always be achieved and refers to preventing all parts involved in the trial (i.e. participants and research personnel) from knowing which allocation the next participant will receive until they have been randomised.  Differently from the ‘sealed envelopes’ method (for which all steps taken to ensure the envelopes will only be opened after the participant is randomised), if the randomisation is performed using a computer-based system (e.g. minimisation) it is reasonable to assume concealment is ensured.  Partially if the mechanism of allocation sequence implementation is described, but is not detailed the steps to conceal the sequence until the interventions are assigned. | [   ] No  [   ] Yes  [   ] Partially |
| 84 | | Consort_Cluster_9 | | **Allocation concealment (cluster trials);** Is it described whether allocation was based on cluster rather than individuals and whether allocation concealment (if any) was at cluster level, individual participant level, or both?  N/A if it is not a cluster trial. | [ ] No  [ ] Yes  [ ] N/A |
| 85 | | Consort_10  Consort_Cluster_10a | | **Allocation implementation:** Who generated the allocation sequence?  The responsible for generating/producing/deriving the random allocation list should be identified. | [   ] No  [   ] Yes |
| 86 | | Consort_10  Consort_Cluster_10a | | **Allocation implementation:** Who enrolled participants (or clusters)?  The responsible for taking the signed informed consent from participants should be clearly identified. | [   ] No  [   ] Yes |
| 87 | | Consort_10  Consort_Cluster_10a | | **Allocation implementation:** Who assigned participants (or clusters) to interventions?  The responsible for randomising the participants should be clearly identified. | [   ] No  [   ] Yes |
| 88 | | Consort_Cluster_10b | | **Allocation implementation (cluster trials);** Is it described the mechanism by which individual participants were included in clusters for the purposes of the trial (such as complete enumeration, random sampling)?  N/A if it is not a cluster trial. | [ ] No  [ ] Yes  [ ] N/A |
| 89 | | Consort_Cluster_10c | | **Allocation implementation (cluster trials);** Is it described from whom consent was sought (representatives of cluster, or individual participants, or both), and whether consent was sought before or after randomisation?  N/A if it is not a cluster trial. | [ ] No  [ ] Yes  [ ] N/A |
| 90 | | Consort_11a | | **Blinding** - Is it detailed whether trial participants were blinded and how?  ('Yes', if both criteria are fulfilled or if a justification is provided when blinding is not possible due to the intervention nature; 'Partially' if not described how blinded if participants were blinded). | [ ] No  [ ] Partially  [ ] Yes |
| 91 | | Consort_11a | | **Blinding** - Is it detailed whether trial care providers were blinded and how?  ('Yes', if both criteria are fulfilled or if a justification is provided when blinding is not possible due to the intervention nature; 'Partially' if not described how blinded if care providers were blinded). | [ ] No  [ ] Partially  [ ] Yes |
| 92 | | Consort_11a | | **Blinding** - Is it detailed whether trial outcomes assessors were blinded and how?  ('Yes', if both criteria are fulfilled; 'Partially' if not described how blinded if outcomes assessors were blinded). | [ ] No  [ ] Partially  [ ] Yes |
| 93 | | Consort_11b | | **Blinding** - Is it described, if relevant, the similarity of interventions to seek evidence of the method of blinding (e.g. appearance, taste, smell, and method of administration)? | [ ] No  [ ] Yes |
| 94 | | Consort_11b | | **Blinding** - Is there a reporting if it was necessary to unblind any participants at any point during the conduct of the trial?  (‘N/A’ for open label trials) | [ ] No  [ ] Yes  [ ] N/A |
| 95 | | Consort_NPT_11c | | **Blinding** - Is there a reporting of any attempts to limit bias if blinding was not possible?  (N/A for open-label trials). | [ ] No  [ ] Yes  [ ] N/A |
| 96 | | Consort_12a | | **Statistical Methods used to compare groups for primary and secondary outcomes** - Is it specified which statistical procedure was used for each analysis? | [ ] No  [ ] Yes |
| 97 | | Consort_Crossover_12a | | **Statistical Methods used to compare groups for primary and secondary outcomes** (crossover trials)- Is it clearly reported how the within participant analysis has been constructed (e.g. using t test on within participant differences, or analysis of variance with participant, period, and treatment effects; or for binary outcomes using conditional logistic regressions)?  N/A if it is not a crossover trial. | [ ] No  [ ] Yes  [ ] N/A |
| 98 | | Consort_Cluster_12a | | **Statistical Methods used to compare groups for primary and secondary outcomes** (cluster trials)- Is it clearly reported how clustering was taken into account?  N/A if it is not a cluster trial. | [ ] No  [ ] Yes  [ ] N/A |
| 99 | | Consort_12a | | **Statistical Methods** -Is it described details of the statistical analysis such as intention-to-treat analysis? | [ ] No  [ ] Yes |
| 100 | | Consort_12a | | **Statistical Methods** - Is it described details of the treatment effect estimate adopted? | [ ] No  [ ] Yes |
| 101 | | Consort_12a | | **Statistical Methods** - Was the treatment effect estimate accompanied by a confidence interval (e.g. 95% CI)? | [ ] No  [ ] Yes |
| 102 | | Consort_12a | | **Statistical Methods** - Are study findings also assessed in terms of their statistical significance (P values)? | [ ] No  [ ] Yes |
| 103 | | Consort_NPT_12a | | **Statistical Methods** - When applicable, do authors provide the details of whether and how clustering by care providers or centres were addressed?  N/A if no clustering by care providers or centres were addressed. | [ ] No  [ ] Yes  [ ] N/A |
| 104 | | Consort_12b | | **Statistical Methods for additional analysis, such as subgroup analysis** - Is there a clear specification of the methods used for subgroup analyses?  Not related to safety. If a statement acknowledging that no additional analyses was conducted, then ‘Yes’. | [ ] No  [ ] Yes |
| 105 | | Consort_12b | | **Statistical Methods for additional analysis, such as adjusted analysis** - Is the rationale and methods used for any adjusted analyses clearly described? Did the authors clarify the choice of variables that were adjusted for, indicate how continuous variables were handled, and specify whether the analysis was planned or suggested by the data?  Not related to safety. If a statement acknowledging that no additional analyses was conducted, then ‘Yes’.  Partially if they described the methods, but did not clarify the choice of variables. | [ ] No  [ ] Yes  [ ] Partially |
| **RESULTS** | | | | | |
| 106 | | Consort_13a  Consort_Cluster_13a | | **Participant’s flow** - Do the authors provide a diagram, including, for each group, the number of participants (or **clusters** who were randomly assigned, received intended treatment, and were analysed for the primary outcome?  Partially if it is not described clearly who were included in these three stages. | [ ] No  [ ] Yes  [ ] Partially |
| 107 | | Consort_NPT_13a | | **Participant’s flow** **(NPT trials)** - Do the authors provide the number of **care providers** or centres performing the intervention in each group and the number of patients treated by each care provider or in each centre, when applicable?  N/A if the intervention was not performed by different centres or care providers - it means, the same team of researchers performed all interventions.  Partially if it is described the number of care providers or centres performing the intervention, but not described the number of patients treated by each of them. | [ ] No  [ ] Yes  [ ] Partially  [ ] N/A |
| 108 | | Consort_Crossover_13a | | **Participant’s flow (crossover trials)** - Do the authors provide a diagram with a clear description of the flow of participants **across periods, using a vertical alignment and including a timescale**?  N/A if it is not a crossover trial. | [ ] No  [ ] Yes  [ ] N/A |
| 109 | | Consort_13a  Consort_SPI_13a | | **Participant’s flow** - Is the number of individuals approached for screening described? | [ ] No  [ ] Yes |
| 110 | | Consort_13a  Consort_SPI_13a | | **Participant’s flow** - Is the number of individuals screened described? | [ ] No  [ ] Yes |
| 111 | | Consort_13a  Consort_SPI_13a | | **Participant’s flow** - Is the number of individuals eligible prior to random assignment provided, with reasons for non-enrolment? (described in the text or diagram) | [ ] No  [ ] Yes |
| 112 | | Consort_13a | | **Participant’s flow** - Is there a description of the number of participants who were randomly assigned for each group? (described in the text or diagram) | [ ] No  [ ] Yes |
| 113 | | Consort_13a | | **Participant’s flow** - Is there a description of the number of participants in each group who received intended treatment and why some participants did not receive the treatment as allocated if it occurred (e.g. were lost to follow-up or excluded from the analysis), if applicable? (described in the text or diagram)  (Partially if the number is described, but it is not described why some participants did not receive the treatment as allocated if it occured) | [ ] No  [ ] Yes  [ ] Partially |
| 114 | | Consort_13a | | **Participant’s flow -** Is there information about whether the investigators included in the analysis all participants who underwent randomisation, in the groups to which they were originally allocated (intention-to treat analysis)? (described in the text or diagram) | [ ] No  [ ] Yes |
| 115 | | Consort_13b | | **Losses and exclusions** - Is it described, for each group, the number of losses and exclusions after randomisation together with reasons (use the term protocol deviation is not sufficient to justify exclusion of participants after randomisation)? (described in the text or diagram) | [ ] No  [ ] Yes |
| 116 | | Consort_13b | | **Losses and exclusions (crossover trials)** - Is it described the loss of participants for each intervention, separately for each sequence and period, with the reasons? (described in the text or diagram)  N/A if it is not a crossover trial. | [ ] No  [ ] Yes  [ ] N/A |
| 117 | | Consort_Cluster_13b | | **Losses and exclusions (cluster trials)** - Is it described the losses and exclusions for both clusters and individual participants? (described in the text or diagram)  N/A if it is not a cluster trial. x | [ ] No  [ ] Yes  [ ] N/A |
| 118 | | Consort_14a | | **Dates defining period of recruitment** - Is it reported? | [ ] No  [ ] Yes |
| 119 | | Consort_14a | | **Dates defining period of follow-up** - Is the period of follow-up reported, including the minimum, maximum, and median duration of follow-up when its length is not a fixed period? | [ ] No  [ ] Yes |
| 120 | | Consort_14b | | **Why the trial ended or was stopped** - Is there a disclosure of the factors extrinsic to the trial that affected the decision to stop the trial, and who decided to stop it, including reporting the role the funding agency played in the deliberations and in the decision to stop the trial?  N/A if the trial was not ended or stopped; Partially if any of the listed information was not described; Yes if all details are presented; No, if trial stopped and none related information is detailed. | [ ] No  [ ] Yes  [ ] Partially  [ ] N/A |
| 121 | | Consort_15  Consort_SPI_15 | | **Baseline characteristics for each group** - Is there a table showing the baseline demographic and clinical characteristics for each group? | [ ] No  [ ] Yes |
| 122 | | Consort_15 | | **Baseline characteristics for each group** - Is it reported the variability of the data for continuous variables, along with average values (e.g. mean and standard deviation or median and a centile range)? | [ ] No  [ ] Yes |
| 123 | | Consort_15 | | **Baseline characteristics for each group** - Were statistical tests applied to check for baseline differences between groups (e.g. P values)? | [ ] No  [ ] Yes |
| 124 | | Consort_Cluster_15 | | **Baseline characteristics for each group (cluster trials)** - Where appliable, are baseline characteristics for cluster and individual participant level presented for each group?  N/A if it is not a cluster study |  |
| 125 | | Consort_NPT_15 | | **Care providers or centres characteristics - When applicable,** is there a description of care providers (qualification, expertise, etc.) and centres in each group?  N/A if the intervention was not provided by different care providers or centres. | [ ] No  [ ] Yes  [ ] N/A |
| 126 | | Consort_16 | | **Number of participants -** Are the number of participants (denominator) included in each analysis reported for each group, and whether the analysis was by original assigned groups? (Yes only if the number of participants per groups is given for ALL analysis). | [ ] No  [ ] Yes |
| 127 | | Consort_Cluster_16 | | **Number of participants (cluster trials)** - Are the number of clusters included in each analysis reported for each group?  N/A if it is not a cluster trial. | [ ] No  [ ] Yes  [ ] N/A |
| 128 | | Consort_16 | | **Number of participants -** For binary outcomes, are the event rates (numerators) also reported or results are presented solely as summary measures, such as relative risks?  ('N/A' if none binary outcome). | [ ] No  [ ] Yes  [ ] N/A |
| 129 | | Consort_16 | | **Intention-to-treat or 'per protocol' analysis** - Were participants analysed according to their original group assignment (intention-to-treat), regardless of what subsequently occurred in the trial? | [ ] No  [ ] Yes |
| 130 | | Consort_17a | | **Treatment effect results** - Are results for each primary outcome presented as the estimated effect size and its precision (such as 95% CI) between groups (treatment effect) ? | [ ] No  [ ] Yes |
| 131 | | Consort_17a  crossover | | **Treatment effect results** - Are results for each primary outcome presented as the estimated effect size and its precision (such as 95% CI) based on within participants comparisons in crossover trials? | [ ] No  [ ] Yes |
| 132 | | Consort_Crossover_17a | | **Treatment effect results (crossover trials)** - Are results for each intervention in each period presented?  N/A if it is not a crossover trial. | [ ] No  [ ] Yes  [ ] N/A |
| 133 | | Consort_Cluster_17a | | **Treatment effect results (cluster trials)** - Are the results presented at cluster or individual participant level, as applicable,  and a coefficient of intracluster correlation (ICC or *k*) for each primary outcome?  N/A if it is not a cluster trial. | [ ] No  [ ] Yes  [ ] N/A |
| 134 | | Consort_17a | | **Treatment effect results** - Are results for all planned primary and secondary outcomes reported and not just for analyses that were statistically significant or interesting? | [ ] No  [ ] Yes |
| 135 | | Consort_17b | | **Treatment effect results** - Are both absolute (risk difference) and relative (relative risk or odds ratio) effect sizes presented for binary outcomes?  ('N/A' if none binary outcome). | [ ] No  [ ] Yes  [ ] N/A |
| 136 | | Consort_18 | | **Treatment effect results** - Are results of any other analyses performed, including subgroup and adjusted analyses, presented?  ('N/A' if none other analyses was performed) | [ ] No  [ ] Yes  [ ] N/A |
| 137 | | Consort_18 | | **Treatment effect results** - If other analyses were performed, were pre-specified distinguished from exploratory ones?  ('N/A' if none other analyses was performed) | [ ] No  [ ] Yes  [ ] N/A |
| 138 | | Consort_18 | | **Treatment effect results** - If subgroups analyses were undertaken, is there a description of which subgroups were examined, why, whether these were pre-specified, and how many were prespecified?  ('N/A' if none other analyses was performed, 'Yes' only if all criteria fulfilled) | [ ] No  [ ] Yes  [ ] Partially  [ ] N/A |
| 139 | | Consort_18 | | **Treatment effect results** - If subgroups analyses were undertaken, is it reported a test of interaction to demonstrate whether the subgroup treatment effects are significantly different from each other?  ('N/A' if none subgroup analyses was performed) | [ ] No  [ ] Yes  [ ] N/A |
| 140 | | Consort_18 | | **Treatment effect results** - If adjusted analyses were undertaken, is there a clear description of the of variables that the analyses were adjust for?  ('N/A' if none adjusted analyses was performed) | [ ] No  [ ] Yes  [ ] N/A |
| 141 | | Consort_18 | | **Treatment effect results** -If adjusted analyses were undertaken, is there a clear description of whether these had been previously described in the trial protocol?  ('N/A' if none adjusted analyses was performed) | [ ] No  [ ] Yes  [ ] N/A |
| *Consort_19* | | | | *Harms or unintended effects - covered by Consort Harms Extension* | [ ] No  [ ] Yes |
| 142 | | Consort_H1 | | **Harms or unintended effects** - Did the study collect data on harms and benefits? Is it stated in the title or abstract?  'Partially' if harm data collected, but not stated in the title or abstract. | [ ] No  [ ] Yes  [ ] Partially |
| 143 | | Consort_H3 | | **Harms or unintended effects** - Is it presented a list addressing adverse events (clinical and laboratory) with definition for each (with attention, when relevant, to grading, expected vs. unexpected events, reference to standardised and validated definitions, and descriptions of new definitions)  Partially if it is presented a list addressing adverse events without definitions for each. | [ ] No  [ ] Yes  [ ] Partially |
| 144 | | Consort_H4 | | **Harms or unintended effects** - Is there a clear description of how harms-related information was collected (mode of data collection, timing, attribution methods, intensity of ascertainment, and harms-related monitoring and stopping rules, if pertinent)?  Partially if an incomplete description of how harms-related information was collected is presented. | [ ] No  [ ] Yes  [ ] Partially |
| 145 | | Consort_H5 | | **Harms or unintended effects** - Is it described in statistical methods how information on harms were analysed? | [ ] No  [ ] Yes |
| 146 | | Consort_H5 | | **Harms or unintended effects** - If harms were major primary or secondary outcomes of the trial, is it described on statistical methods any formal statistical analyses rather than descriptive statistics? | [ ] No  [ ] Yes  [ ] N/A |
| 147 | | Consort_H6 | | **Harms or unintended effects** - the number of participant’s withdrawals that are due to harms and the experience with the allocated treatment described as results, for each arm?  N/A if no withdrawals. | [ ] No  [ ] Yes  [ ] N/A |
| 148 | | Consort_H7 | | **Harms or unintended effects** - Is the denominator for analyses on harms described in the results for each arm? | [ ] No  [ ] Yes |
| 149 | | Consort_H8 | | **Harms or unintended effects** - Is the absolute risk of each adverse event, specifying type, grade, and seriousness per arm described in results for each arm? (If no adverse events of a specific type and severity occurred, it should so state). | [ ] No  [ ] Yes |
| 150 | | Consort_H9 | | **Harms or unintended effects** - Is any subgroup or exploratory analyses for harms described in Rasults? | [ ] No  [ ] Yes |
| **DISCUSSION** | | | | | |
| 151 | | Consort_H10 | | **Harms or unintended effects** - Is a balanced discussion of benefits and harms with emphasis on study limitations, generalizability, and other sources of information presented for harms? | [ ] No  [ ] Yes |
| 152 | | Consort_20 | | **Trial limitations -** Are the trial limitations discussed, including addressing sources of potential bias? | [ ] No  [ ] Yes |
| 153 | | Consort_20 | | **Trial limitations -** Are the trial limitations discussed, including addressing potential imprecisions, such as measurement error of a primary outcome or diagnosis (e.g. scale used, assessor not trained)? | [ ] No  [ ] Yes |
| 154 | | Consort_20 | | **Trial limitations -** Are the trial limitations discussed, including addressing multiplicity of analyses, if relevant?  N/A if multiple analyses were not performed. | [ ] No  [ ] Yes  [ ] N/A |
| 155 | | Consort_20 | | **Trial limitations -** Are the difference between significance and clinical importance of trial's findings considered by authors in the Discussion Section? | [ ] No  [ ] Yes |
| 156 | | Consort_Crossover_20 | | **Trial limitations (crossover trials) -** Are the potential carry over effects considered by authors in the Discussion Section ?  N/A if it is not a crossover trial. | [ ] No  [ ] Yes  [ ] N/A |
| 157 | | Consort_NPT_20 | | **Trial limitations -** Are the trial limitations discussed taking into account the choice of comparator, lack of partial blind, or unequal expertise of care providers or centres in each group, when applicable? | [ ] No  [ ] Yes |
| 158 | | Consort_21  Consort_NPT_21 | | **Generalisability of trials findings -** Is there a discussion on the external validity and applicability of the trial findings, according to the interventions, comparators, patients, and care providers and centres involved in the trial, when applicable? | [ ] No  [ ] Yes |
| 159 | | Consort_Cluster_21 | | [ ] No  [ ] Yes  [ ] N/A | [ ] No  [ ] Yes  [ ] N/A |
| 160 | | Consort_22 | | **Interpretation consistent with results -** Is there an interpretation consistent with results, balancing benefits and harms? | [ ] No  [ ] Yes |
| 161 | | Consort_22 | | **Interpretation consistent with results -** Is there an interpretation consistent with results, considering other relevant evidence based on a comprehensive search, rather than being limited to studies that support the results of the current trial (e.g. including a systematic review of similar trials)? | [ ] No  [ ] Yes |
| **OTHER INFORMATION** | | | | | |
| 162 | | Consort_23 | **Registration number and name of trial register** - Are the register number and name of trial register provided? (If authors had not registered their trial they should explicitly state this and give the reason) | | [ ] No  [ ] Yes |
| 163 | | Consort_24 | **Full trial protocol -** Is it described where the full trial protocol can be accessed, if available? | | [ ] No  [ ] Yes |
| 164 | | Consort_SPI_17a | | **Indicate availability of trial data -** is it indicated how data can be accessed?  (Authors should indicate whether and how to obtain trial datasets, including any metadata and analytic code needed to replicate the reported analyses. Any legal or ethical restrictions on making the trial data available should be described) | [ ] No  [ ] Yes |
| 165 | | Consort_25 | **Funding and other support -** Are the sources of funding and other support described? | | [ ] No  [ ] Yes |
| 166 | | Consort_25 | **Funding and other support -** Is the role of funders described? | | [ ] No  [ ] Yes |
| 167 | | Consort_SPI_25b | **Declaration of any other potential interests -** In addition to financial interests, do the authors declare any other potential interests that may be perceived to influence the design, conduct, analysis, or reporting of the trial following established criteria (i.e. . allegiance to or professional training in evaluated interventions). | | [ ] No  [ ] Yes |
| 168 | | Consort_SPI_new_a | **Stakeholder involvement -** Is it described whether intervention developers were involved in designing the trial, delivering the intervention, assessing the outcomes, or interpreting the data?  N/A if the intervention is not related to nutrition education or counselling. | | [ ] No  [ ] Yes  [ ] N/A |
| 169 | | Consort_SPI_new_b | **Other Stakeholder involvement -** Is it described whether other stakeholders were involved in the trial?  (When applicable, authors should describe which stakeholders were involved, how they were recruited, and how they were involved in various stages of the trial)  N/A if the intervention is not related to nutrition education or counselling. | | [ ] No  [ ] Yes  [ ] N/A |
| 170 | | Consort_SPI_new_c | **Incentives offered as part of the trial -** Is it described whether any incentives were offered as part of the trial? (Authors should describe the type of incentives, at what trial stage and for what purpose incentives are offered, and what these incentives entail. Authors also should state whether incentives differ by trial group).  N/A if no incentive was offered. | | [ ] No  [ ] Yes  [ ] Partially |
