# Supplementary material for: Completeness of Reporting in Diet- and Nutrition-Related Randomized Controlled Trials and Systematic Reviews With Meta-Analysis: Protocol for 2 Independent Meta-Research Studies
Source: JMIR Res Protoc. 2023 Mar 23;12:e43537. doi: 10.2196/43537 (PMC10131600; doi:10.2196/43537)
Supplement: Multimedia Appendix 5 [file resprot_v12i1e43537_app5.docx]

**Multimedia Appendix 5. Standardized form for data extraction for systematic reviews with meta-analyses of diet- or nutrition-related RCTs published as scientific articles in peer-reviewed journals.**

*Publication and meta-analyses features*

1. Study ID: ______

2. First author:_________________

3. Number of authors: _________

4. Journal: __________________

5. PMID/ DOI: ________________________________

6. PICOS:

6.1.1. Participants:

( ) Pregnant women ( ) Mother and infant pairs

( ) Infants ( ) Children and preschool-aged children

( ) Adults ( ) Elderly

( ) Adults and elderly ( ) Postmenopausal women

( ) Participants with a clinical condition (s) (specify it)____________

6.1.2. Participants:

Cancer ( ) yes ( ) no

Cardiovascular disease ( ) yes ( ) no

6.2. Intervention:

( ) Food (whole food, food products, specially formulated foods)

( ) Breastfeeding, complementary feeding.

( ) Complete diet or dietary patterns

( ) Complete nutrition formulas (enteral or parenteral)

( ) Supplementation or supplements (single or multiple nutrients, bioactive non-nutrients, plant components)

( ) Nutrition education, counseling and coordination of care

( ) Other, if no component of intervention could be categorized as any of the above (specify it)____________

6.3. Comparator:

( ) Placebo

( ) No intervention

( ) Usual care

( ) Different intervention

( ) Other (specify it)____________

6.4. Primary outcome:

( ) Not specified

( ) Mortality

( ) Clinical status (clinical or biochemical measures)

( ) Nutritional status (anthropometry, body composition, nutrition diagnosis)

( ) Frequency or severity of disease

( ) Diet quality and/or variety

( ) Food/ nutrient/ dietary intake

( ) Diet-related behaviors

( ) Other non-dietary behaviors

( ) Withdrawal from the study, drop-out or adherence-related

( ) Adverse events, side-effects and/or safety

( ) Cost-effectiveness or economic

( ) Quality of life

( ) Breastfeeding

( ) Other (specify it)____________

6.5.1 Primary Studies design:

( ) parallel RCT ( ) crossover RCT

( ) cluster RCT ( ) non-cluster RCT

( ) factorial ( ) non-factorial

( ) two arms ( ) multi-arms

( ) unicentric ( ) multicentric

7. Registered protocol:

( ) Yes ( ) PROSPERO ( ) Other

( ) No

8. Number of studies included in the systematic review:________

9. Number de studies included in the meta-analysis of primary outcome:________

10. Risk of bias of primary studies:

( ) evaluated ( ) ROB2 ( ) other tool ______________

( ) not evaluated

11. Certainty of evidence of systematic review:

( ) evaluated by GRADE approach

( ) evaluated by other approach

( ) not evaluated

*Presence of spin*

|  | Main text | Abstract |
| --- | --- | --- |
| Misleading reporting | 1) Failure to acknowledge a departure from protocol that could modify the interpretation of results  ( ) yes ( ) no | 1) Selective reporting of or overemphasis on efficacy outcomes favoring the beneficial effect of the experimental intervention ( ) yes ( ) no |
|  | 2) Selective reporting of or overemphasis on efficacy outcomes favoring the beneficial effect of the experimental intervention (e.g., secondary outcomes, subgroup analyses)  ( ) yes ( ) no | 2) Selective reporting of or overemphasis on harm outcomes favoring the safety of the experimental intervention  ( ) yes ( ) no |
|  | 3) Selective reporting of or overemphasis on harm outcomes favoring the safety of the experimental intervention  ( ) yes ( ) no | 3) Failure to report a wide confidence interval of estimates ( ) yes ( ) no |
|  | 4) No or inadequate reporting of the limitations of the systematic review  ( ) yes ( ) no | 4) Authors hide or do not present any conflict of interest  ( ) yes ( ) no |
|  | 5) Selective citation of articles in favor of the beneficial effect of the experimental intervention  ( ) yes ( ) no | 5) Inadequate focus on the results of primary studies favoring the beneficial effect of the experimental intervention instead of the meta-analysis results  ( ) yes ( ) no |
|  | 6) Authors hide or do not present any conflict of interest  ( ) yes ( ) no | 6) Conclusion focusing selectively on statistically significant efficacy outcome  ( ) yes ( ) no |
|  | 7) Conclusion focusing selectively on statistically significant efficacy outcome  ( ) yes ( ) no | 7) Failure to report the number of studies/patients actually contributing to the analysis for main outcomes  ( ) yes ( ) no |
|  | 8) Selective reporting of analysis favoring the beneficial effect of the experimental intervention (e.g., selective analysis using random or fixed effect according the results)  ( ) yes ( ) no | 8) Failure to specify the direction of the effect when it favors the control intervention  ( ) yes ( ) no |
|  | 9) Inadequate focus on the results of primary studies favoring the beneficial effect of the experimental intervention instead of the meta-analysis results  ( ) yes ( ) no |  |
|  | 10) Changing the scale of the forest plot to magnify the results (diamond size)  ( ) yes ( ) no |  |
| Misleading interpretation | 11) Title claims or suggests a beneficial effect of the experimental intervention not supported by the findings  ( ) yes ( ) no | 9) Title claims or suggests a beneficial effect of the experimental intervention not supported by the findings  ( ) yes ( ) no |
|  | 12) Inadequate interpretation of non- statistically significant results (with a wide confidence interval) as a lack of effect or an equivalent effect for efficacy outcomes  ( ) yes ( ) no | 10) Inadequate focus on *p* value instead of magnitude of the effect estimates for harm or efficacy outcome  ( ) yes ( ) no |
|  | 13) Inadequate interpretation of non- statistically significant results (with a wide confidence interval) as demonstrating safety for harm outcome  ( ) yes ( ) no | 11) Focus on relative effect when the  absolute effect is small  ( ) yes ( ) no |
|  | 14) Inadequate focus on *p* value instead of magnitude of the effect estimates for harm or efficacy outcome  ( ) yes ( ) no | 12) Conclusion claiming equivalence or comparable effectiveness for non- statistically significant results with a wide confidence interval  ( ) yes ( ) no |
|  | 15) Focus on relative effect when the absolute effect is small  ( ) yes ( ) no | 13) Conclusion formulating recommendations for clinical practice not supported by the findings  ( ) yes ( ) no |
|  | 16) Misleading interpretation of cited articles, favoring the beneficial effect of the experimental intervention  ( ) yes ( ) no | 14) Conclusion claiming safety based on non-statistically significant results with a wide confidence interval  ( ) yes ( ) no |
|  | 17) Conclusion claiming equivalence or comparable effectiveness for non-statistically significant results with a wide confidence interval  ( ) yes ( ) no | 15) Conclusion claiming the beneficial effect of the experimental treatment despite high risk of bias in primary studies  ( ) yes ( ) no |
|  | 18) Conclusion formulating recommendations for clinical practice not supported by the findings  ( ) yes ( ) no | 16) Conclusion claiming the beneficial effect of the experimental treatment despite reporting bias  ( ) yes ( ) no |
|  | 19) Conclusion claiming safety based on non-statistically significant results with a wide confidence interval  ( ) yes ( ) no | 17) Conclusion claiming the beneficial effect of the experimental treatment despite high heterogeneity  ( ) yes ( ) no |
|  | 20) Conclusion ignoring the high risk of bias of the studies, the heterogeneity or the reporting bias (i.e., a low level of evidence) in the interpretation of the results  ( ) yes ( ) no | 18) Ignoring that the review included different study design (e.g., controlled trial or observational studies  ( ) yes ( ) no |
|  | 21) No or inadequate consideration of heterogeneity in results interpretation (i.e., no assessment of heterogeneity reported, claiming the absence of heterogeneity not supported by the data, claiming the beneficial effect of the treatment despite high heterogeneity, no downgrading the evidence in cases of high heterogeneity, interpreting non-statistical significant results for the test of heterogeneity as an evidence of no heterogeneity etc.)  ( ) yes ( ) no |  |
|  | 22) No or inadequate consideration of the risk of bias of primary studies included in results interpretation (i.e., no reporting of the risk of bias of the primary studies, claiming the low risk of bias of studies included not supported by the data, no downgrading the evidence despite several high risk of bias studies)  ( ) yes ( ) no |  |
|  | 23) No or inadequate consideration of  reporting bias in results  interpretation (i.e., no reporting of  an assessment of reporting bias,  claiming efficacy despite an  evidence of reporting bias,  claiming the absence of reporting  bias not supported by the data,  negative test result interpreted as  absence of publication bias, use of  the test without the condition of validity, inadequate interpretation  of a funnel plot, etc.)  ( ) yes ( ) no |  |
| Inappropriate extrapolation | 24) Inadequate extrapolation of the results from surrogate markers or specific outcome to the global improvement of the disease  ( ) yes ( ) no | 19) Conclusion extrapolating the review’s findings to a different population or setting  ( ) yes ( ) no |
|  | 25) Inadequate extrapolation of the results to a larger population, a larger setting or a wider set of interventions (e.g., from a specific rehabilitation program to all rehabilitation programs, to a specialized unit to a non- specialized medical unit etc.)  ( ) yes ( ) no | 20) Conclusion extrapolating the review’s findings to a different intervention (i.e., claiming efficacy of one specific intervention although the review covers a class of several interventions)  ( ) yes ( ) no |
|  | 26) Conclusion extrapolating the review’s findings to a different population or setting | 21) Conclusion extrapolating the review’s findings from a surrogate marker or a specific outcome to the global improvement of the disease  ( ) yes ( ) no |
|  | 27) Conclusion extrapolating the review’s findings to a different intervention (i.e., claiming efficacy of one specific intervention although the review covers a class of several interventions)  ( ) yes ( ) no |  |
|  | 28) Conclusion extrapolating the review’s findings from a surrogate marker or a specific outcome to the global improvement of the disease  ( ) yes ( ) no |  |
