# Supplementary material for: Completeness of Reporting in Diet- and Nutrition-Related Randomized Controlled Trials and Systematic Reviews With Meta-Analysis: Protocol for 2 Independent Meta-Research Studies
Source: JMIR Res Protoc. 2023 Mar 23;12:e43537. doi: 10.2196/43537 (PMC10131600; doi:10.2196/43537)
Supplement: Multimedia Appendix 6 [file resprot_v12i1e43537_app6.docx]

**Multimedia Appendix 6. Data extraction form: completeness reporting of a recent sample of meta-analyses of nutrition or diet-related RCTs published in peer-reviewed journals.**

| **Section and Topic** | |  | **Item #** | **Checklist item** | | | **Reporting** |
| --- | --- | --- | --- | --- | --- | --- | --- |
|  | **TITLE** | | | | | |  |
| Title | | 1 | 1 | Identify the report as a systematic review with meta-analysis. | | | ( ) Yes  ( ) No |
|  | **ABSTRACT** | | | | | |  |
| Abstract | | 2 | 2_P-A | Provide an explicit statement of the main objective(s) or question(s) the review addresses. | | | ( ) Yes  ( ) No |
|  |  | 3 | 3_P-A | Specify the inclusion and exclusion criteria for the review. | | | ( ) Yes  ( ) No |
|  |  | 4 | 4_P-A | Specify the information sources (e.g. databases, registers) used to identify studies and the date when each was last searched. | | | ( ) Yes  ( ) No |
|  |  | 5 | 5_P-A | Specify the methods used to assess risk of bias in the included studies. | | | ( ) Yes  ( ) No |
|  |  | 6 | 6_P-A | Specify the methods used to present and synthesize results. | | | ( ) Yes  ( ) No |
|  |  | 7 | 7_P-A | Give the total number of included studies and participants | | | ( ) Yes  ( ) No |
|  |  | 8 | 7_P-A | Summarize relevant characteristics of studies.  (describe what we mean by relevant characteristics PICOS) | | | ( ) Yes  ( ) No |
|  |  | 9 | 8_P-A | Present results for main outcomes, in terms of summary estimate and confidence/credible interval. | | | ( ) Yes  ( ) No |
|  |  | 10 | 8_P-A | Indicate the number of included studies and participants for each meta-analysis performed.. | | | ( ) Yes  ( ) No |
|  |  | 11 | 8_P-A | Indicate the direction of the effect (i.e. which group is favored). | | | ( ) Yes  ( ) No |
|  |  | 12 | 9_P-A | Provide a brief summary of the (any) limitations of the evidence included in the review (e.g. study risk of bias, inconsistency and imprecision). | | | ( ) Yes  ( ) No |
|  |  | 13 | 10_P-A | Provide a general interpretation of the results  (Does the conclusion provide an answer to the research question/study objective?) | | | ( ) Yes  ( ) No |
|  |  | 14 | 10_P-A | Do the authors mention the implications of the MA findings? | | | ( ) Yes  ( ) No |
|  | **INTRODUCTION** | | | | | |  |
| Rationale | | 15 | 3 | Describe the rationale for the review in the context of existing knowledge.  (Why was the review done?) | | | ( ) Yes  ( ) No |
| Objectives | | 16 | 4 | Provide an explicit statement of the objective(s) or question(s) the review addresses.  (What does the review aim?) | | | ( ) Yes  ( ) No |
|  | **METHODS** | | | | | |  |
| Eligibility criteria | | 17 | 5 | | Specify the inclusion and exclusion criteria for the review  (Partially if specified inclusion or exclusion criteria, but not both) | | ( ) Yes  ( ) No  ( ) Partially |
|  |  | 18 | 5* | | Specify the inclusion and exclusion criteria for the review - Participants  (accept as 'yes' statements that no study was excluded based on participants characteristics) | | ( ) Yes  ( ) No |
|  |  | 19 | 5* | | Specify the inclusion and exclusion criteria for the review - Intervention description | | ( ) Yes  ( ) No |
|  |  | 20 | 5* | | Specify the inclusion and exclusion criteria for the review - Comparator | | ( ) Yes  ( ) No |
|  |  | 21 | 5* | | Specify the inclusion and exclusion criteria for the review - Outcomes | | ( ) Yes  ( ) No |
|  |  | 22 | 5* | | Specify the inclusion and exclusion criteria for the review - RCT design  (accept as 'yes' statements that no study was excluded based on RCT design) | | ( ) Yes  ( ) No |
|  |  | 23 | 5* | | Specify the inclusion and exclusion criteria for the review - Intervention duration  (accept as 'yes' statements that no study was excluded based on intervention duration) | | ( ) Yes  ( ) No |
|  |  | 24 | 5 | | Specify how studies were grouped for the syntheses | | ( ) Yes  ( ) No |
| Information sources | | 25 | 1_P-S | | Name each individual database searched, stating the platform for each.  (Partially if the platform is not stated) | | ( ) Yes  ( ) No  ( ) Partially |
|  |  | 26 | 2_P-S | | If databases were searched simultaneously on a single platform, state the name of the platform, listing all of the databases searched.  (Partially if the platform is stated, but the databases were not listed; N/A if a single platform is not mentioned) | | ( ) Yes  ( ) No  ( ) Partially  ( ) N/A |
|  |  | 27 | 3_P-S | | List any study registries searched.  (N/A if clearly described that study registries were not searched) | | ( ) Yes  ( ) No  ( ) N/A |
|  |  | 28 | 4_P-S | | Describe any online or print source purposefully searched or browsed (e.g., tables of contents, print conference proceedings, web sites), and how this was done.  (Partially if they are reported, but it is not described how this was done) | | ( ) Yes  ( ) No  ( ) Partially |
|  |  | 29 | 5_P-S | | Indicate whether cited references or citing references were examined, and describe any methods used for locating cited/citing references (e.g., browsing reference lists, using a citation index, setting up email alerts for references citing included studies).  (Partially if cited references were checked, but it is not described the methods used for this) | | ( ) No  ( ) Yes  ( ) Partially |
|  |  | 30 | 6_P-S | | Indicate whether additional studies or data were sought by contacting authors, experts, manufacturers, or others. | | ( ) Yes  ( ) No |
|  |  | 31 | 7_P-S | | Describe methods for searching any additional information sources.  (N/A if no further search sources mentioned) | | ( ) Yes  ( ) No  ( ) N/A |
| Search strategy | | 32 | 8_P-S | | Include the search strategies for each database and information source, copied and pasted exactly as run. | | ( ) Yes  ( ) No |
|  |  | 33 | 9_P-S | | Specify that no limits were used, or describe any limits or restrictions applied to a search (e.g., date or time period, language, study design) | | ( ) Yes  ( ) No |
|  |  | 34 | 9_P-S | | Provide justification for using/not using limits in the search.  (N/A if no limits were reported) | | ( ) Yes  ( ) No  ( ) N/A |
|  |  | 35 | 10_P-S | | Indicate whether published search filters were used (as originally designed or modified), and if so, cite the filter(s) used.  (Partially if a published search filter was used, but it was not cited) | | ( ) Yes  ( ) No  ( ) Partially |
|  |  | 36 | 11_P-S | | Indicate when search strategies from other literature reviews were adapted or reused for a substantive part or the entire search, citing the previous review(s).  (N/A when no mention of other literature reviews related to search strategies). | | ( ) Yes  ( ) No  ( ) N/A |
|  |  | 37 | 12_P-S | | Report the methods used to update the search(es) (e.g., rerunning searches, email alerts).  (N/A when no mention to update of search) | | ( ) Yes  ( ) No  ( ) N/A |
|  |  | 38 | 13_P-S | | For each search strategy, provide the date when the last search occurred. | | ( ) Yes  ( ) No |
|  |  | 39 | 14_P-S | | Describe any search peer review process. | | ( ) Yes  ( ) No |
| Selection process | | 40 | 8 | | Specify the methods used to decide whether a study met the inclusion criteria of the review | | ( ) Yes  ( ) No |
|  |  | 41 | 8 | | Specify how many reviewers screened each record and each report retrieved, | | ( ) Yes  ( ) No |
|  |  | 42 | 8 | | Specify whether reviewers screened each record independently  (N/A if just one reviewer screened each record) | | ( ) Yes  ( ) No  ( ) N/A |
|  |  | 43 | 8 | | Specify, if applicable, details of automation tools used in the selection process.  (N/A if clearly described that no automation tool was used). | | ( ) Yes  ( ) No  ( ) N/A |
| Data collection process | | 44 | 9 | | Specify the methods used to collect data from reports | | ( ) Yes  ( ) No |
|  |  | 45 | 9 | | Specify how many reviewers collected data from each report. | | ( ) Yes  ( ) No |
|  |  | 46 | 9 | | Specify whether reviewers collected data from each report independently.  (N/A if just one reviewer collected data from each report) | | ( ) Yes  ( ) No  ( ) N/A |
|  |  | 47 | 9 | | Specify any processes for obtaining or confirming data from study investigators  (N/A if no data was sought from original studies’ investigators) | | ( ) Yes  ( ) No  ( ) N/A |
|  |  | 48 | 9 | | Specify, if applicable, details of automation tools used in the process of data collection.  (N/A if clearly described that no automation tool was used). | | ( ) Yes  ( ) No  ( ) N/A |
| Data items | | 49 | TIDieR_1 | | Interventions description: Provide the name or a phrase that describes the intervention (s) included in the review, as defined by the review authors. | | [   ] No  [   ] Yes |
|  |  | 50 | 10a | | List and define all outcomes for which data were sought, as defined by the review authors. | | ( ) Yes  ( ) No |
|  |  | 51 | 10a | | Specify whether all results that were compatible with each outcome domain in each study were sought (e.g. for all measures, time points, analyses), and if not, the methods used to decide which results to collect. | | ( ) Yes  ( ) No |
|  |  | 52 | 10b | | List and define all other variables for which data were sought (e.g. participant and intervention characteristics, funding sources). | | ( ) Yes  ( ) No |
|  |  | 53 | 10b | | Describe any assumptions made about any missing or unclear information. | | ( ) Yes  ( ) No |
| Study risk of bias assessment | | 54 | 11 | | Specify the methods used to assess risk of bias in the included studies, including details of the tool(s) used  (Partially if no details about the tools or instruments of assessment are provided) | | ( ) Yes  ( ) No  ( ) Partially |
|  |  | 55 | 11 | | Specify how many reviewers assessed risk of bias in each study | | ( ) Yes  ( ) No |
|  |  | 56 | 11 | | Specify whether the reviewers assessed risk of bias in each study independently  (N/A if just one reviewer assessed the risk of bias in each study) | | ( ) Yes  ( ) No  ( ) N/A |
|  |  | 57 | 11 | | Specify, if applicable, details of automation tools used in the study risk of bias assessment. . | | ( ) Yes  ( ) No  ( ) N/A |
| Effect measures | | 58 | 12 | | Specify for each outcome the effect measure(s) (e.g. risk ratio, mean difference) used in the synthesis or presentation of results. | | ( ) Yes  ( ) No |
| Synthesis methods | | 59 | 13a | | Describe the processes used to decide which studies were eligible for each synthesis (e.g. tabulating the study intervention characteristics and comparing against the planned groups for each synthesis (item #5)). | | ( ) Yes  ( ) No |
|  |  | 60 | 13b | | Describe any methods required to prepare the data for presentation or synthesis, such as handling of missing summary statistics, or data conversions. | | ( ) Yes  ( ) No |
|  |  | 61 | 13c | | Describe any methods used to tabulate or visually display results of individual studies and syntheses. | | ( ) Yes  ( ) No |
|  |  | 62 | 13d | | Describe any methods used to synthesize results and provide a rationale for the choice(s).  (Partially if no rationale provided) | | ( ) Yes  ( ) No  ( ) Partially |
|  |  | 63 | 13d | | Describe the model(s) used in the meta- analysis  (i.e. random or fixed effects models) | | ( ) Yes  ( ) No |
|  |  | 64 | 13d | | Describe the method(s) applied to identify the presence and extent of statistical heterogeneity  (e.g. *I*2; Chromba’s test) | | ( ) Yes  ( ) No |
|  |  | 65 | 13d | | Describe the software package(s) used for the meta-analyses. | | ( ) Yes  ( ) No |
|  |  | 66 | 13e | | Describe any methods used to explore possible causes of heterogeneity among study results (e.g. subgroup analysis, meta-regression). | | ( ) Yes  ( ) No |
|  |  | 67 | 13f | | Describe any sensitivity analyses conducted to assess robustness of the synthesized results.  (N/A if no sensitivity analyses reported) | | ( ) Yes  ( ) No  ( ) N/A |
| Reporting bias assessment | | 68 | 14a | | Describe any methods used to assess risk of bias in primary studies included in the review. | | ( ) Yes  ( ) No |
|  |  | 69 | 14b | | Describe any methods used to assess risk of publication bias in the review. | | ( ) Yes  ( ) No |
| Certainty assessment | | 70 | 15 | | Describe any methods used to assess certainty (or confidence) in the body of evidence for an outcome. | | ( ) Yes  ( ) No |
|  | **RESULTS** | | | | | |  |
| Study selection | | 71 | 15_P-S | | | Document the total number of records identified from each database and other information sources. | ( ) Yes  ( ) No |
|  |  | 72 | 16_P-S | | | Describe the processes and any software used to deduplication records from multiple database searches and other information sources. | ( ) Yes  ( ) No |
|  |  | 73 | 16a | | | Describe the results of the search and selection process, from the number of records identified in the search to the number of studies included in the review. | ( ) Yes  ( ) No |
|  |  | 74 | 16a | | | Describe the results of the search and selection process using a flow diagram. | ( ) Yes  ( ) No |
|  |  | 75 | 16b | | | Cite studies that might appear to meet the inclusion criteria, but which were excluded, and explain why they were excluded.  (Partially if the reason for exclusion was not reported). | ( ) Yes  ( ) No  ( ) Partially |
| Study characteristics | | 76 | 17 | | | Cite each included study and present its characteristics (ideally detailed characteristics of PICOTS approach - participants, intervention, comparator, outcomes, duration and study design) | ( ) Yes  ( ) No |
|  | | 77 | TIDIeR_1 | | | Present for each study the details of the intervention, as defined by the included studies’ authors.  (Is it provided the name or a phrase that describes the intervention (s)?)  ‘Partially’ if described for some interventions categories, but not for all | ( ) Yes  ( ) No  ( ) Partially |
|  | | 78 | TIDIeR_3 | | | Present for each study the details of the intervention - providers, as defined by the included studies’ authors.  (Is it described, for each category of intervention, who was the intervention(s) provider(s) (e.g. psychologist, nursing assistant)?)  ‘Partially’ if described for some interventions categories, but not for all | ( ) Yes  ( ) No  ( ) Partially |
|  | | 79 | TIDIeR_6 | | | Present for each study the details of the intervention - delivery mode, as defined by the included studies’ authors.  (Are the modes of delivery (e.g. face-to-face or by some other mechanism, such as internet or telephone) of the intervention(s) described?)  ‘Partially’ if described for some interventions categories, but not for all | ( ) Yes  ( ) No  ( ) Partially |
|  | | 80 | TIDIeR_6 | | | Present for each study the details of the intervention - delivery structure, as defined by the included studies’ authors.  (Is it described whether the intervention(s) was provided individually or in a group?)  ‘Partially’ if described for some interventions categories, but not for all | ( ) Yes  ( ) No  ( ) Partially |
|  | | 81 | TIDIeR_8 | | | Present for each study the details of the intervention - quantity, as defined by the included studies’ authors.  (Is it described the number of times (e.g. one time or 5 sessions) the intervention(s) was delivered?)  ‘Partially’ if described for some interventions categories, but not for all | ( ) Yes  ( ) No  ( ) Partially |
|  | | 82 | TIDIeR_8 | | | Present for each study the details of the intervention - frequency, as defined by the included studies’ authors.  (Is it described the frequency (e.g. every two weeks or daily) with which the intervention(s) was delivered?)  ‘Partially’ if described for some interventions categories, but not for all | ( ) Yes  ( ) No  ( ) Partially |
|  | | 83 | TIDIeR_8 | | | Present for each study the details of the intervention - period, as defined by the included studies’ authors.  (Is it described the period (e.g. 6 months) over which the intervention(s) was delivered? In crossover trials, this must include information about any run-in and washout periods, or a statement on why these are not needed.)  ‘Partially’ if described for some interventions categories, but not for all | ( ) Yes  ( ) No  ( ) Partially |
|  | | 84 | TIDIeR_8 | | | Present for each study the details of the intervention - amount, as defined by the included studies’ authors.  (Is it described the amount (length of session, or dose) of the intervention that was delivered at each time?)  ‘Partially’ if described for some interventions categories, but not for all | ( ) Yes  ( ) No  ( ) Partially |
|  | | 85 | TIDIeR_11 | | | Present for each study the details of the intervention - adherence planned, as defined by the included studies’ authors.  (Is it described how the intervention adherence was assessed? (e.g., drug tablet return, laboratory tests)? | ( ) Yes  ( ) No |
|  | | 86 | TIDIeR_12 | | | Present for each study the details of the intervention - adherence actual as observed in each included study.  (Is it described the extent to which the intervention was delivered as planned?)  ‘Partially’ if described for some interventions categories, but not for all | ( ) Yes  ( ) No  ( ) Partially |
| Risk of bias in studies | | 87 | 18 | | | Present assessments of risk of bias for each included study. | ( ) Yes  ( ) No |
| Results of individual studies | | 88 | 19 | | | For all outcomes, present (a) summary statistics for each group in each study, ideally using structured tables or plots. | ( ) Yes  ( ) No |
|  |  | 89 | 19 | | | For all outcomes, present: (b) an effect estimate and its precision (e.g. confidence/credible interval) for each included study, ideally using structured tables or plots. | ( ) Yes  ( ) No |
| Results of syntheses | | 90 | 20a | | | For each synthesis, provide a brief summary of the characteristics of contributing studies.  (Yes if already if provided in a table with data of all studies included in the review) | ( ) Yes  ( ) No |
|  |  | 91 | 20a | | | For each synthesis, briefly summarise the risk of bias among contributing studies. | ( ) Yes  ( ) No |
|  |  | 92 | 20b | | | For each synthesis, present the summary estimate and its precision (e.g. confidence/credible interval) | ( ) Yes  ( ) No |
|  |  | 93 | 20b | | | For each synthesis, present the summary estimate the measures of statistical heterogeneity.. | ( ) Yes  ( ) No |
|  |  | 94 | 20b | | | If comparing groups, describe the direction of the effect of each measure of heterogeneity. | ( ) Yes  ( ) No |
|  |  | 95 | 20c | | | Present results of all investigations of possible causes of heterogeneity among study results.  (N/A if authors reported no significant heterogeneity to be explored). | ( ) Yes  ( ) No  ( ) N/A |
|  |  | 96 | 20d | | | Present results of all sensitivity analyses conducted to assess the robustness of the synthesized results.  (Yes if authors justified why these analysis were not performed - e.g. reduced number of studies) | ( ) Yes  ( ) No |
| Reporting biases | | 97 | 21 | | | Present assessments of risk of bias in primary studies included in the review. | ( ) Yes  ( ) No |
|  | | 98 | 21 | | | Present assessments of risk of publication bias in the review. (Yes if the authors justified that this evaluation was not performed due to the reduced number of primary studies) | ( ) Yes  ( ) No |
| Certainty of evidence | | 99 | 22 | | | Present assessments of certainty (or confidence) in the body of evidence for each outcome assessed. | ( ) Yes  ( ) No |
|  | **DISCUSSION** | | | | | |  |
| Discussion | | 100 | 23a | Provide a general interpretation of the results in the context of other evidence. | | | ( ) Yes  ( ) No |
|  |  | 101 | 23b | Discuss any limitations of the evidence included in the review. | | | ( ) Yes  ( ) No |
|  |  | 102 | 23c | Discuss any limitations of the review processes used. | | | ( ) Yes  ( ) No |
|  |  | 103 | 23d | Discuss implications of the results for practice, policy, and future research. | | | ( ) Yes  ( ) No |
|  | **OTHER INFORMATION** | | | | | |  |
| Registration and protocol | | 104 | 24a | Provide registration information for the review, including register name and registration number, or state that the review was not registered.  (Partially if the register name or register number is provided, but not both) | | | ( ) Yes  ( ) No  ( ) Partially |
|  |  | 105 | 24b | Indicate where the review protocol can be accessed, or state that a protocol was not prepared. | | | ( ) Yes  ( ) No |
|  |  | 106 | 24c | Describe and explain any amendments to information provided at registration or in the protocol.  (N/A if clearly stated that the original protocol was not amended.) | | | ( ) Yes  ( ) No  ( ) N/A |
| Support | | 107 | 25 | Describe sources of financial or non-financial support for the review | | | ( ) Yes  ( ) No |
|  |  | 108 | 25 | Describe the role of the funders or sponsors in the review.  (N/A if clearly described that there were no funders or sponsors) | | | ( ) Yes  ( ) No  ( ) N/A |
| Competing interests | | 109 | 26 | Declare any competing interests of review authors. | | | ( ) Yes  ( ) No |
| Availability of data, code and other materials | | 110 | 27 | Report if template data collection forms are publicly available and where they can be found.  (Partially if it was not described where they can be found) | | | ( ) Yes  ( ) No  ( ) Partially |
|  |  | 111 | 27 | Report if data extracted from included studies are **publicly available** and where they can be found.  (Partially if available but not described where they can be found) | | | ( ) Yes  ( ) No  ( ) Partially |
|  |  | 112 | 27 | Report if data used for all analyses are **publicly available** and where they can be found.  (Partially if available but not described where they can be found) | | | ( ) Yes  ( ) No  ( ) Partially |
|  |  | 113 | 27 | Report if analytic code is **publicly available** and where they can be found.  (Partially if available but not described where it can be found) | | | ( ) Yes  ( ) No  ( ) Partially |
|  |  | 114 | 27 | Report if any other materials used in the review are **publicly available** and where they can be found.  (Partially if available but not described where they can be found) | | | ( ) Yes  ( ) No  ( ) Partially |
